# Phylogenomics of trophically diverse cichlids disentangles processes driving adaptive radiation and repeated trophic transitions

**DOI:** 10.1002/ece3.9077

**Published:** 2022-07-17

**Authors:** Pooja Singh, Iker Irisarri, Julián Torres‐Dowdall, Gerhard G. Thallinger, Hannes Svardal, Emily Moriarty Lemmon, Alan R. Lemmon, Stephan Koblmüller, Axel Meyer, Christian Sturmbauer

**Affiliations:** ^1^ Institute of Biology University of Graz Graz Austria; ^2^ Lehrstuhl für Zoologie und Evolutionsbiologie, Department of Biology University of Konstanz Constance Germany; ^3^ Institute of Ecology and Evolution University of Bern Bern Switzerland; ^4^ Leibniz Institute for the Analysis of Biodiversity Change (LIB), Zoological Museum Hamburg Hamburg Germany; ^5^ Institute of Biomedical Informatics Graz University of Technology Graz Austria; ^6^ OMICS Center Graz, BioTechMed Graz Graz Austria; ^7^ Department of Biology University of Antwerp Antwerp Belgium; ^8^ Naturalis Biodiversity Center Leiden The Netherlands; ^9^ Department of Biological Science Florida State University, Biomedical Research Facility Tallahassee Florida USA

**Keywords:** adaptive radiation, carnivory, cichlid, herbivory, trophic adaptation

## Abstract

Cichlid fishes of the tribe Tropheini are a striking case of adaptive radiation, exemplifying multiple trophic transitions between herbivory and carnivory occurring in sympatry with other established cichlid lineages. Tropheini evolved highly specialized eco‐morphologies to exploit similar trophic niches in different ways repeatedly and rapidly. To better understand the evolutionary history and trophic adaptations of this lineage, we generated a dataset of 532 targeted loci from 21 out of the 22 described Tropheini species. We resolved the Tropheini into seven monophyletic genera and discovered one to be polyphyletic. The polyphyletic genus, *Petrochromis*, represents three convergent origins of the algae grazing trophic specialization. This repeated evolution of grazing may have been facilitated by adaptive introgression as we found evidence for gene flow among algae grazing genera. We also found evidence of gene flow among algae browsing genera, but gene flow was restricted between herbivorous and carnivorous genera. Furthermore, we observed no evidence supporting a hybrid origin of this radiation. Our molecular evolutionary analyses suggest that opsin genes likely evolved in response to selection pressures associated with trophic ecology in the Tropheini. We found surprisingly little evidence of positive selection in coding regions of jaw‐shaping genes in this trophically diverse lineage. This suggests low degrees of freedom for further change in these genes, and possibly a larger role for regulatory variation in driving jaw adaptations. Our study emphasizes Tropheini cichlids as an important model for studying the evolution of trophic specialization and its role in speciation.

## Graphical abstract

Trophic adaptations and transitions are important features of diversification. Our study emphasizes the importance of Tropheini cichlids as a model for studying the evolution of trophic transitions and their role in speciation using an anchored phylogenomics approach. We show how gene flow can contribute to repeated and rapid trophic evolution.

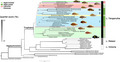

## 1 INTRODUCTION

Adaptive radiation is the proliferation of an ancestral species into an array of lineages adapted to a variety of ecological niches (Schluter, 2000). The radiating species often evolve a key innovation enabling them to outcompete other species (Stroud & Losos, 2020). Along with Darwin's Finches (Lack, 1947) and *Anolis* lizards (Losos et al., 1997), cichlid fishes from the East African Great Lakes are one of the best‐known examples of adaptive radiation (Fryer & Iles, 1972). Cichlids were by far the most successful lineage whose diversification was linked to geological and climatic changes shaping lake habitats all over East Africa. A unique aspect of even very young cichlid radiations is evolution of trophic diversity characterized by repeated transitions between herbivory and carnivory, from an ancestor that was most likely an omnivore or “generalist” (McGee et al., 2020). Such trophic transitions to herbivory have not been observed among postglacial fish radiations (Seehausen & Wagner, 2014) or other relatively young freshwater radiations (Schluter et al., 1996). This makes cichlids an excellent system to study the evolution of trophic adaptations in general, and at short timeframes.

An important cichlid radiation that is aptly named for its successful diversification in a subset of littoral trophic niche space is the Tropheini radiation from Lake Tanganyika (LT) (Wanek & Sturmbauer, 2015; Yamaoka, 1983a, 1983b). The current understanding is that the Tropheini are the earliest extant lacustrine offshoot of the lineage leading to modern haplochromines, which also includes the speciose cichlid radiations of Lake Malawi and Victoria. Lake Tanganyika is situated in the East African rift and it has central, north, and south basins that began to form 9–12 Ma, 7–8 Ma and 2–4 Ma, respectively (Cohen et al., 1993). A re‐colonization of the deepening littoral zone of LT was proposed as the origin of the Tropheini ~5–7 Ma (Irisarri et al., 2018; Ronco et al., 2021). This hypothesis purports that the ancestor of the Tropheini entered the lake environment in intense competition established species from six non‐haplochromine lineages that are endemic to LT. The diversification of the Tropheini has been attributed to extrinsic factors such as the abovementioned deepening lacustrine conditions as well as lake‐level fluctuations (Sefc, Mattersdorfer, Hermann, et al., 2017; Sturmbauer, 1998; Sturmbauer et al., 2001; Sturmbauer & Meyer, 1992, 1993). Currently, 22 described species (Fricke et al., 2022) and several undescribed species (Konings, 2019; Ronco et al., 2020) comprise the Tropheini and they hold a key ecological position in the rock and cobble shore habitats of LT (Koblmüller et al., 2010; Sturmbauer et al., 2003; Takahashi & Koblmüller, 2014). Several of these species form complex arrays of geographic races and sister species (Egger et al., 2007; Konings, 2019; Van Steenberge et al., 2018).

Morphologically, the nine described Tropheini genera are distinguished on the basis of the mouth shape, oral dentition, and pharyngeal jaw shape—all strong indicators for diet and foraging (Poll, 1986). Tropheini entered three distinct carnivorous niches, and repeatedly entered highly specialized browser and grazer subniches several times (Sturmbauer et al., 2003). The tribe is comprised of (i) three genera of differentially specialized carnivores (*Ctenochromis*, *Gnathochromis*, and *Lobochilotes*) that inhabit shallow, sedimented habitats and feed on invertebrates and small fish (Konings, 2019); (ii) two genera (*Petrochromis*, *Interochromis*) of algae grazers that comb unicellular algae and detritus (Yamaoka, 1997); (iii) three genera of algae browsers (*Tropheus*, *Simochromis*, *Pseudosimochromis*) that bite off epilithic algae (Yamaoka, 1997); and (iv) the omnivorous genus *Limnotilapia*. Both algae browser and algae grazer species inhabit shallow and rocky or partially rocky habitats and are, with the exception of the monotypic *Interochromis* and *Simochromis*, species‐rich compared to the monotypic carnivore and omnivore genera. *Lobochilotes*, *Simochromis*, *Interochromis*, *Limnotilapia*, *Gnathochromis*, and *Ctenochromis* are monotypic genera, while *Tropheus*, *Petrochromis*, and *Pseudosimochromis* comprise several species.

The trophic novelty of Tropheini species is not just related to the diversity of food they eat, such as algae, invertebrates, zooplankton, juvenile fish; but they have also evolved different ways to eat the same type of food source. This is especially true for algae‐feeding, as the majority of Tropheini species are epilithic algae feeders (Poll, 1986). For example, species of the genus *Petrochromis* are algae grazers combing filamentous algae with their elongated tricuspid teeth; and species of the genus *Tropheus* browse algae using their bicuspid teeth with a continuous cutting edge to bite off algae (Yamaoka, 1983a, 1983b). This makes the Tropheini an important model to understand evolutionary transitions between herbivory and carnivory, and how these transitions contribute to adaptive radiation.

The phylogenetic relationships among Tropheini species and the conditions under which they diversified are still not fully resolved (Koblmüller et al., 2010; Ronco et al., 2021; Sturmbauer et al., 2003). Previous phylogenetic studies, which used mitochondrial DNA (Sturmbauer et al., 2003) and AFLPs (Koblmüller et al., 2010), could not fully resolve the phylogenetic relationships among the Tropheini. Ronco et al. (2021) used whole genome sequencing data to construct the phylogeny of the Tropheini as part of the larger LT radiation, but several key branches were not resolved with high support. The aim of our study is (1) to investigate the evolutionary dynamics of trophic specialization at the intra and intergeneric levels within the Tropheini using a robust phylogenetic framework based on an independent dataset to previously published studies and (2) to evaluate the extent of incomplete lineage sorting (ILS) and hybridization, which are characteristics of adaptive radiation. We also study molecular evolutionary patterns of candidate genes associated with jaw morphology (linked to diet) and two other important adaptations of the Tropheini: vision (linked to diet and sexual selection) and body color (linked to sexual selection).

## 2 MATERIALS AND METHODS

### 2.1 Sample collection and anchored loci sequencing

Specimens for this study were collected between 2004 and 2015 at various sampling locations in LT or obtained via the ornamental fish trade. 21 out of 22 described species (Fricke et al., 2022) from all major lineages of the Tropheini are included in this study, only *Pseudosimochromis margaretae* is missing. In addition, some yet undescribed *Tropheus* species as well as *Petrochromis* of the *P. polyodon* species complex (with yet unclear species status) are included. Tissue samples were preserved in >95% ethanol (see File S1 for details of species names, sampling site, and sampling year). Phylogenomic data were generated at the Centre for Anchored Phylogenetics using the methodology detailed below (www.anchoredphylogeny.com) (Lemmon et al., 2012). DNA was extracted from fin clips, sheared with a Covaris E220 Focused‐ultrasonicator, and indexed libraries were prepared on a Beckman–Coulter Biomek FXp liquid‐handling robot. Indexed samples were pooled at equal quantities (12–16 samples per pool) and enriched, after which equimolar quantities were pooled and sequenced in paired‐end, 150‐bp mode on Illumina HiSeq 2000. Paired overlapping reads were merged, sequencing error corrected, and adapters trimmed. Reads were assembled using a quasi de novo approach in an in‐house pipeline (Hamilton et al., 2016) and orthologous sets were identified by sequence similarity clustering and neighbor‐joining‐like process. Anchored loci correspond to those developed by (Stout et al., 2016), which target exons from 260 gene families specifically designed for teleosts. In addition, we supplemented the bait set with 29 genes with functions related to color vision (*lws*, *rh2a‐α*, *rh2a‐ß*, *rh2b*, *sws1*, *sws2a*, *sws2b*) (Carleton, 2009), coloration (*csf1ra*, *dlc*, *fbxo36b*, *hagaromo*, *kir7.1*, *kir7.2*, *kita*, *kitla*, *mitfa*, *smtlb*, *sox10*) (Dutton et al., 2001; Fukamachi et al., 2009; Hamada et al., 2014; Henning et al., 2014; Kawakami et al., 2000; Masafumi et al., 2012; McGill et al., 2002; Miller et al., 2007; Parichy et al., 1999; Salzburger et al., 2007; Singh et al., 2014; Terai et al., 2003; Watanabe et al., 2007) and jaw (bone and tooth) formation (*barx1*, *bmp2*, *bmp4*, *c‐fos*, *col6a1*, *creb1*, *dlx2*, *pitx2*, *runx2b*, *shh*, *sp7*) (Betancur et al., 2010; Fraser et al., 2009; Gunter et al., 2013; Schneider et al., 2014) (File S4a).

### 2.2 Dataset assembly and alignment details

We removed 9 out of 541 loci because of suspected paralogy (see Irisarri et al., 2018): one locus (KCNJ13) produced BLAST hits in two regions of the *O. niloticus* genome and has been identified as duplicated (Brawand et al., 2014); the remaining seven loci had a high proportion (>3%) of ambiguous base calls, suggesting that consensus sequences might be assembled from reads originating from paralogous loci. As an additional step to check for correct orthology, we calculated pairwise genetic distances for all sequences in each locus and excluded the presence of further paralogs because all genetic distances were very low (<0.05%) and congruent with the expected species divergences. The final data set contained 532 anchored loci for 51 individuals, which were concatenated into a matrix of 966,914 aligned nucleotide positions. Since we aimed to specifically study the phylogenetic relationships and evolutionary and dynamics within the tribe Tropheini, its taxon sampling was significantly expanded compared to Irisarri et al. (2018) (32 vs. 16 Tropheini individuals).

In addition to the newly sequenced taxa, five species were added to the dataset from available cichlid genomes, *Serranochromis* sp. “checkerboard,” *Haplochromis paucidens* and *Orthochromis* sp. “red cheek” (McGee et al., 2016) and *Tropheus moorii* and *Petrochromis trewavasae* (Fischer et al., 2021). Orthologous loci in these genomes were identified as best hits by BLASTN using the longest sequence per loci as query, and hit coordinates were extended by 500 bp upstream and downstream to capture the less conserved regions flanking exons. BEDtools (Quinlan & Hall, 2010) was used to extract the FASTA sequences from the genomes taking reading frame orientation into account and the correct orthology of new sequences was confirmed with reciprocal BLASTN searches against the original anchored loci, discarding any nonreciprocal hits.

Sequences were aligned with MAFFT v.7245 (Katoh & Standley, 2013) with settings “‐ep 0 –genafpair –maxiterate 1000” (similar to E‐INS‐i) and positions with >80% gaps were removed using trimAl v.1.4 (“‐gt 0.2”) (Capella‐Gutierrez et al., 2009). All alignments were manually curated.

### 2.3 Phylogenomic analyses

A concatenated maximum likelihood tree was constructed with RAxML v. 7.3.1 (Stamatakis, 2006) and branch support assessed by 500 pseudoreplicates of nonparametric bootstrapping (“‐f a ‐m GTRCAT ‐N 500”). A summary coalescent species tree was estimated with ASTRAL‐II v5.6.3 (Mirarab & Warnow, 2015) with default parameters, using maximum likelihood gene trees generated with IQ‐TREE v1.3.13 (Nguyen et al., 2014) and 120 site‐bootstrap resampling, using independent best‐fit model parameters per gene (“iqtree‐omp ‐quiet ‐s input.fas ‐st NT ‐nt 1 ‐m TEST ‐b 120”). Branch support for the coalescent species tree was assessed by local posterior probabilities (Sayyari & Mirarab, 2016) and 100 replicates of multilocus bootstrapping with site resampling. Branch‐specific normalized quartet scores (i.e., proportion of quartets in gene trees that agree with that branch) generated by ASTRAL‐II were extracted as a measure of gene tree discordance.

### 2.4 Time‐calibrated phylogeny

Due to the lack of appropriate fossils to date this tree, we used secondary node calibrations from two previous studies (Irisarri et al., 2018; Matschiner et al., 2017). Our secondary calibration schemes (S01, S02, S03) relied on estimates of Irisarri et al. (2018) based on vicariance calibration of Gondwana fragmentation (S01), a fossil‐only calibration using cichlid fossils (*Mahengechromis* sp., *Sarotherodon martini*, *Oreochromis lorenzoi*, *Tylochromis* and *Tugenchromis pickfordi*) (S02), and a fossil‐only calibration scheme that included upper bounds for vicariance nodes (S03). Our calibration scheme S04 was based on age estimates from Matschiner et al. (2017), who used cichlid and non‐cichlid fossil calibrations and a model of time‐homogeneous diversification and fossilization processes, from which prior densities for clade ages were derived given the age of the oldest fossil record of each clade.

All four secondary calibration schemes used the minimum and maximum ages (95% confidence interval) of two calibration nodes (i) the split between Haplochromini and *Orthochromis uvinzae* and (ii) the split between *Astatoreochromis* and Lake Victoria + Lake Malawi + *Astatotilapia* burtoni. (Table 1). Molecular dating was performed with RelTime (Tamura et al., 2012) as implemented in MEGA‐CC v.7.0.2078, using the RAxML phylogeny as input tree. Dating assumed the “local clocks” model, applying the GTR + Γ with five gamma categories (the prevalent model for single loci identified with jModeltest (Posada, 2008) and the branch swap filter was set to “None” and fixed the topology inferred by RAxML (Figure S1).

**TABLE 1:** Calibration schemes used to obtain divergence time estimates

Calibration scheme	Calibration node	Min age	Max age	Source
S01	Haplochromini/*Orthochromis uvinzae* split	3.07	12.74	Estimates obtained with calibration Scheme C06 (vicariance dependent), from Irisarri*, Singh* et al. (2018) based on Gondwana fragmentation
	Astatoreochromis/LV + LM + Astatotilapia split	1.35	7.91	
S02	Haplochromini/*Orthochromis uvinzae* split	2.43	14.17	Estimates obtained with calibration Scheme C07 (fossil only) from Irisarri*, Singh* et al. (2018) based on *Mahengechromis* sp., *Sarotherodon martini* and *Oreochromis lorenzoi*, *Tylochromis* and *Tugenchromis pickfordi* fossils
	Astatoreochromis/LV + LM + Astatotilapia split	0.89	8.84	
S03	Haplochromini/*Orthochromis uvinzae* split	4.28	12.60	Estimates obtained with calibration Scheme C10 (fossil‐only like but including upper bound for vicariance node) from Irisarri*, Singh* et al. (2018
	Astatoreochromis/LV + LM + Astatotilapia split	2.10	7.79	
S04	Haplochromini/*Orthochromis uvinzae* split	28.36	44.54	Estimates with calibration scheme from Matschiner et al. (2017
	Astatoreochomis/LV + LM + Astatotilapia split	18.25	39.39	

*Note*: Each scheme includes the same two calibration nodes. Minimum and maximum ages are in million years ago (ma) and in all cases relevant references are indicated.

### 2.5 Estimating speciation/extinction rates

RPANDA (Morlon et al., 2010) was used to fit different models that vary in the speciation/extinction rates to study Tropheini diversification. RPANDA tests complex macroevolutionary models where speciation/extinction rates can vary across time, in contrast with the more commonly used time‐homogeneous models. Specifically, the following models were tested: (1) constant speciation; no extinction (2) constant speciation, constant extinction; (3) exponential variation of speciation rate across time, no extinction (4) linear variation of speciation rate across time, no extinction (5) exponential variation of speciation rate, constant extinction (6) linear variation speciation rate, constant extinction (7) exponential variation of speciation & extinction rates (8) linear variation of speciation and extinction rates. The different models were fitted with the “fit_bd” function in RPANDA, using time‐calibrated phylogenies of the Tropheini inferred with S01, S02, S03 secondary calibration schemes (S04 was not considered), after excluding all non‐Tropheini taxa and duplicated conspecifics with ape's “drop.tip” function (Paradis & Schliep, 2019).

### 2.6 F‐statistics

**FIGURE 1:**
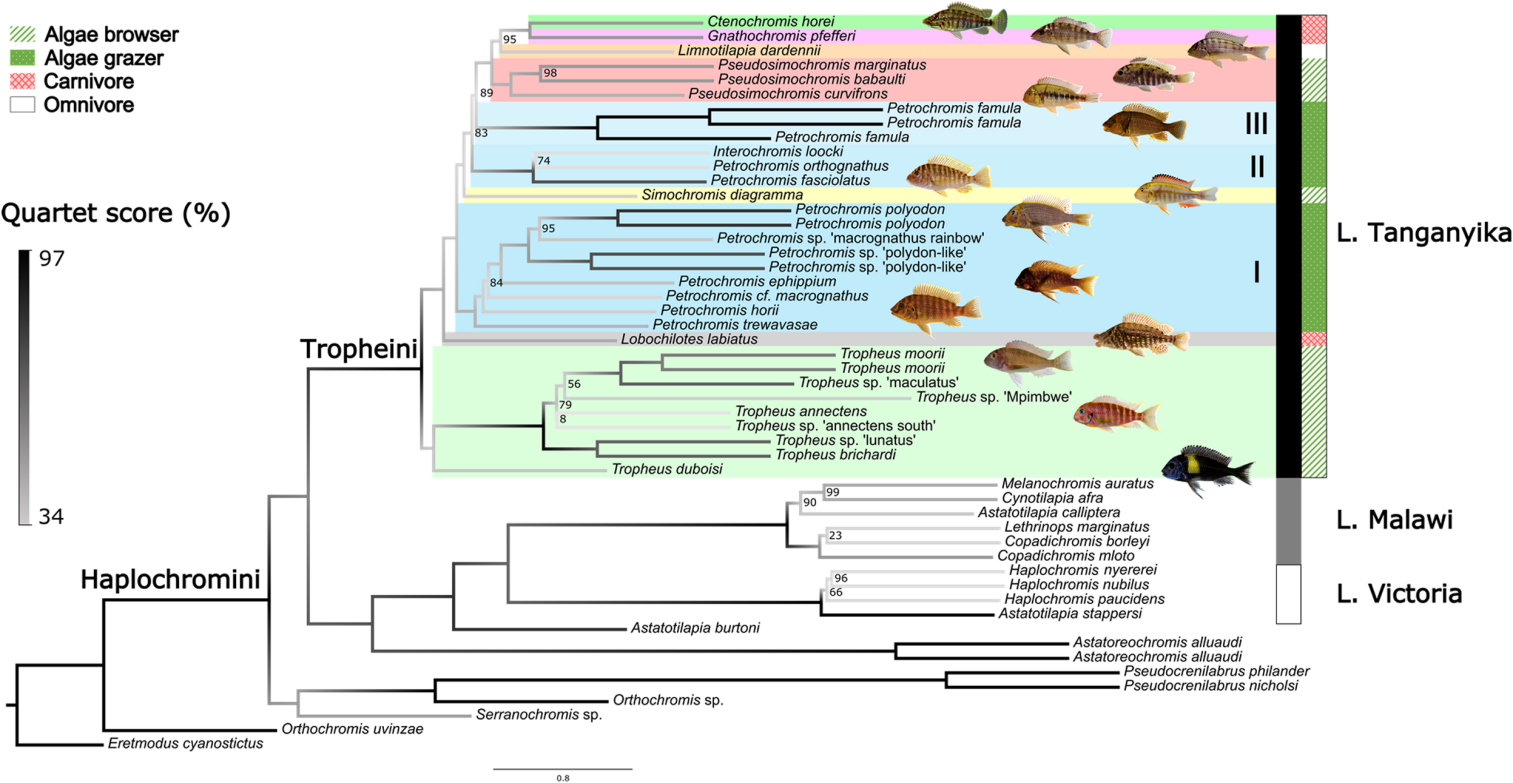
ASTRAL summary coalescent tree of the Tropheini. Numbers at nodes are multilocus bootstrap proportions; only values lower than 100% are shown. Branch lengths are in coalescent units and branch colors depict quartet scores as a measure of gene‐tree discordance, with lighter colors indicating higher discordance. The colored rectangles depict assignment to different Tropheini genera. *Petrochromis* is divided into clades I, II, and III. Interochromis shares many features with *Petrochromis* and is nested deeply within them, therefore no separate color was used. Trophic adaptations are annotated. NB: *Orthochromis* genus is polyphyletic where *Orthochromis* sp. “red cheek” is from the tribe haplochromine and *Orthochromis uvinzae* is from the tribe Orthochromini

F‐branch is a recently developed summary of F4 admixture ratio tests (a type of ABBA/BABA statistic; [Patterson et al., 2012]) that is able to identify patterns of allele sharing between nonsister branches of a phylogeny that are consistent with gene flow (Malinsky et al., 2020). To apply F‐branch to the phylogenetic groups identified in Figure 1, we first converted the multi‐FASTA supermatrix to VCF format using snp‐sites (https://github.com/sanger‐pathogens/snp‐sites) with the option v. A custom script was used to update the header, set the genotypes to “0/0” and “1/1” instead of 0 and 1 as required by Dsuite, remove multiallelic sites, and set deletions as missing genotypes (‘./.’). The latter is necessary to retain sites where one of the samples did not align. The F4‐ratio was calculated using Dsuite with the command line ‘Dsuite Dtrios ‐t tree.newick ‐‐JKnum 10 input.vcf tropheini_groups_SETS.txt’ for the groups defined in in Figure 1 and *E. cyanostictus* as outgroup. We used the output of this to compute f‐branch using the functions dstat.get_fmin_tree and dstat.get_branch_mat available in (https://github.com/feilchenfeldt/pypopgen3). For this, we only retained F4‐ratio values with an associated block‐jackknife *p*‐value < .05 as calculated by Dsuite. All other F4‐ratios were set to 0.

### 2.7 Molecular evolutionary analysis

To investigate signatures of selection of genes associated with key traits (color vision, body coloration, and jaw morphology), we included genes implicated in these three traits in previous studies using functional and transcriptomic analyses (see File S4a for details). The sequences containing premature stop codons within the ORF were excluded and exons from the same gene were concatenated into a single alignment after CDSs were extracted according to the reference coordinates in the *O. niloticus* genome (see File S4b for loci‐gene correspondence). Opsin genes *rh2a‐α* and *rh2a‐ß* share a high sequence identity and the assembly pipeline produced alignments in which reads coming from both paralogs were mixed and thus were excluded. Evidence of molecular evolution in the functional loci alignments were tested using random site models in PAML (Yang, 2007). First, we used Model 0 as implemented in PAML to estimate overall (i.e., across all sites and species) dN/dS ratio for each of the 27 genes considered to be functionally important, as well all the other loci in our data set (File S4a). Then, we tested for variation in the dN/dS ratio across sites (M3/M0), and for the presence of positively selected sites (M2a/M1a) by comparing the likelihood of alternative site models with the likelihood ratio tests (LRTs).

Using clade model C (CmC) in PAML, we tested three specific hypotheses for the evolution of the 27 genes: (1) divergence in the rate of molecular evolution between Tropheini and the other species in our alignment, (2) divergence in the rate of molecular evolution between sexually monochromatic and sexually dichromatic cichlid species, and (3) divergence in the rate of molecular evolution among cichlid species with different feeding modes (generalists, grazers, browsers). CmC allows some sites to vary among a priori defined groups of species (background vs. foreground branches). The significance for CmC models was tested against a null model of neutral evolution (M2a_rel) (Weadick & Chang, 2011) using LRTs, followed by correcting for multiple hypothesis tests using the Benjamini–Hochberg procedure (FDR = 0.05). The relative fit of the CmC models assuming different data partitions according to the trait of interest was measured by differences in AIC values. In order to define taxa partitions objectively for color dimorphism and feeding modes, we reconstructed ancestral states for these two character using the re‐rooting method as implemented in the R package phytools (Revell, 2012). To minimize the number of transitions and avoid stochastic errors in single gene trees, ancestral reconstructions used the concatenated RAxML phylogeny considering only changes with a marginal posterior probability > .85.

## 3 RESULTS

### 3.1 Phylogenomic resolution of the Tropheini

The coalescent ASTRAL phylogeny of 21 described Tropheini species (File S1) based on 532 loci (966,914 bp total alignment) was fully resolved and most branches received high statistical support (90% of nodes had >75% bootstrap support) (Figure 1). The first two splits within the haplochromines concern two mainly riverine lineages that do not occur in LT: *Orthochromis*/*Serranochromis*, as representatives of a lineage that is found from the Lower Congo River to Southern Africa, and *Pseudocrenilabrus*, which is distributed in rivers from Northern to Southern Africa. The Tropheini were divided into seven monophyletic genera (of which five are monotypic) and one polyphyletic genus. The *Tropheus* species complex, a genus of algae browsers inhabiting rocky shores, is sister group to all other Tropheini. This is followed by a deep split between *Lobochilotes labiatus*, an invertebrate feeder with large lips and fleshy lobes, and the remaining genera. *Petrochromis*, a polyphyletic genus of algae grazers is recovered in three separate lineages (Clades I, II, III). It must be noted that *Interochromis loocki* is recovered in *Petrochromis* Clade II and *I. loockii* is known among aquarists as *Petrochromis* sp. “orthognathus tricolor” (Konings, 1998). *Simochromis diagramma*, an algae browser, is recovered between *Petrochromis* clade I and II. *Petrochromis* clade III is the sister group to the remaining Tropheini: the algae browsing genus *Pseudosimochromis*, the omnivore *Limnotilapia dardennii* and the two highly specialized and distinct carnivores *Gnathochromis pfefferi* and *Ctenochromis horei*. Our dataset missed one described Tropheini species, *P. margaretae*, but its absence is unlikely to affect the deeper splits in our Tropheini phylogeny, and thus it would not affect the divergence time estimates.

The concatenated tree obtained with RAxML was strongly supported (96% of nodes had >75% bootstrap support; Figure S1) and recovered a similar topology to the coalescent ASTRAL tree. These two trees differed in 15 out of 51 branches (~29%), of which only 11% were in Tropheini (Figure S1) and none of these concerned deepest splits that are relevant for estimating divergence times. Differently resolved branches had generally low support. For the Tropheini, the main differences between the concatenated phylogeny and the coalescent species tree were (1) within the *Tropheus* species complex, (2) within *Petrochromis* clade I, and (3) within *Petrochromis* clade II. While some incongruences may result from limited phylogenetic resolution of our data, other incongruences such as the relationships between *I. loocki*, *P. fasciolatus* and *P. orthognathus* (Petrochromis Clade II), or the relationship between *Tropheus moorii* Mpimbwe and *T. annectens*—might be indicative of ILS or recent clade‐internal hybridization. The extent of hybridization and ILS is discussed in more detail in the next sections.

### 3.2 Timing of Tropheini evolution and speciation rates

**FIGURE 2:**
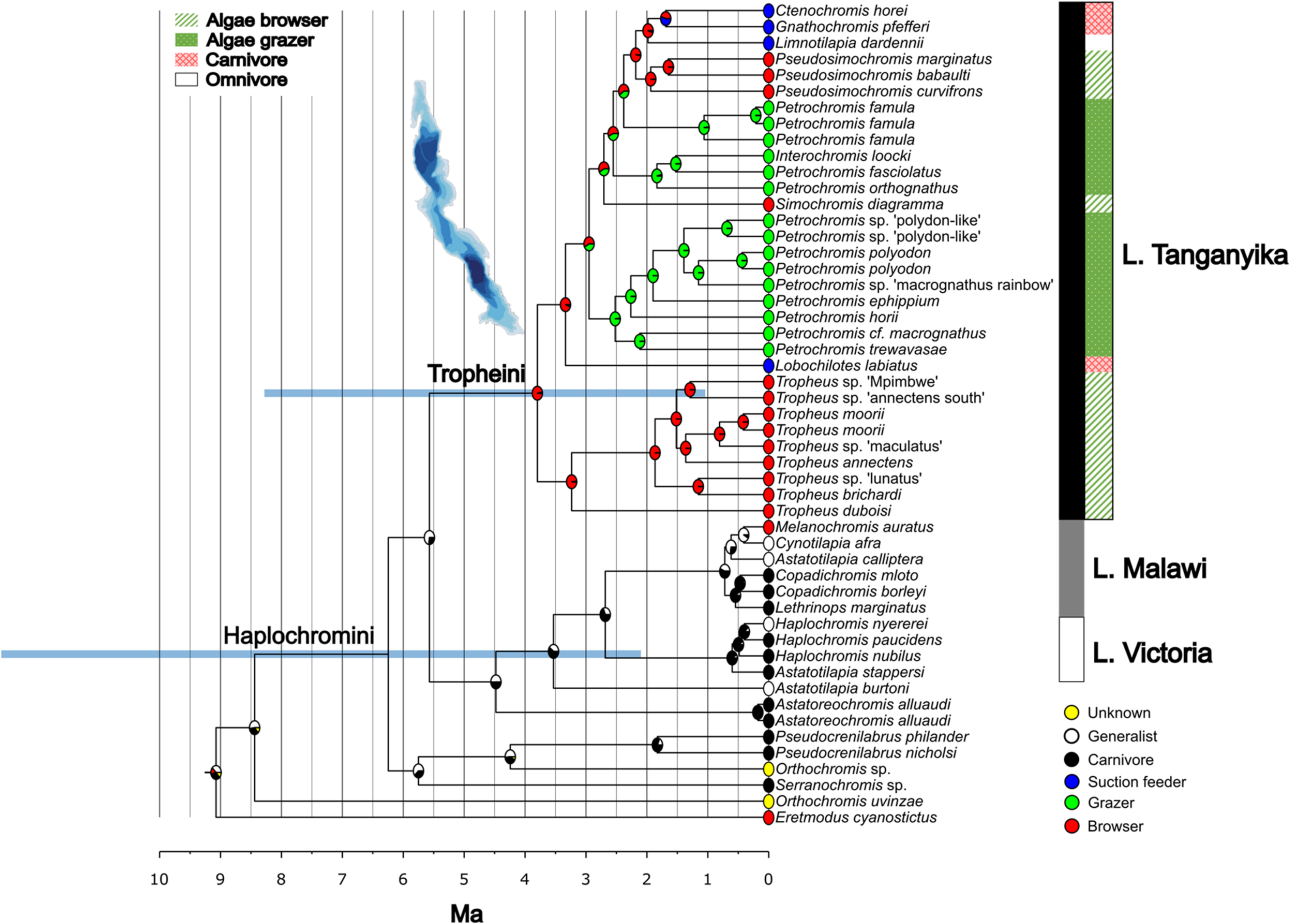
Ancestral state reconstruction of trophic specializations and time calibrated RAxML phylogeny of Tropheini (confidence intervals of key nodes marked by blue boxes). Divergence times have been inferred with RelTime with S03 secondary calibration scheme. X‐axis scale is in million years ago (Ma). Detailed information of the divergence times can be found in Table 1 and File S2

To obtain divergence times for the Tropheini radiation, we used four different secondary time calibration schemes based on two phylogenomic studies, which produced markedly different time estimates for cichlids (Table 1, Figures 2 and S2, File S2, Methods). These differences derive from the different ages used as secondary calibrations in the four schemes. One of the schemes (S01) assumed early cichlid divergences to co‐occur with Gondwana fragmentation (Irisarri et al., 2018), two schemes were based on calibrations exclusively from cichlid fossils (S02, S03) (Irisarri et al., 2018), and the fourth scheme (S04) used age estimates inferred by applying fossil calibrations of non‐cichlid fishes (Matschiner et al. (2017). Differences in age estimates of Tropheini between S03 and S01 and S02 were small. The S03 calibration scheme produced the most convincing divergence time estimates based on the paleolimnological history of Lake Tanganyika: The split between the Tropheini and other haplochromines was 5.6 Ma (95% confidence interval 2.1–11.8 Ma) and the age of the Tropheini was estimated at 3.8 Ma (95% confidence interval 1.0–8.3 Ma), followed by emergence of *Tropheus* lineage at 3.2 Ma (95% confidence interval 0.7–7.5 Ma) (Figure 2). S01 estimated the age of the Tropheini at 3.6 Ma (0.7–8.4 Ma) and S02 estimated the age at 3.7 Ma (0.6–9.3 Ma). S02 fossil calibration scheme produced slightly larger 95% confidence intervals than S01. The age estimate for the Tropheini based on the fourth calibration scheme (S04) was 17.8 Ma (11.2–25.5 Ma) and the estimated ages for the split between other haplochromines and the Tropheini was 26.0 Ma (18.3–36.0 Ma), with the emergence of *Tropheus* at 15.1 Ma (8.7–22.8 Ma).

To gain insight into the diversification patterns of the Tropheini, we fit different time‐homogeneous and time‐heterogeneous macroevolutionary diversification models, finding support for a linear decrease of speciation rate and no extinction (ΔAICc = 2.54 to the next model of linear variation of speciation rate and constant extinction; Figure S3). The obtained results are consistent across the S01, S02, and S03 calibration schemes. The S04 time‐calibration was not included in this analysis as its age estimates for the split of the Haplochromine/Orthochromini and the Eretmodini/Orthochromini were too old (> 40 Ma) to be realistic with regard to the formation of Lake Tanganyika, which began to form between 12 and 9 Ma (Cohen et al., 1993).

### 3.3 Incomplete lineage sorting and hybridization

**FIGURE 3:**
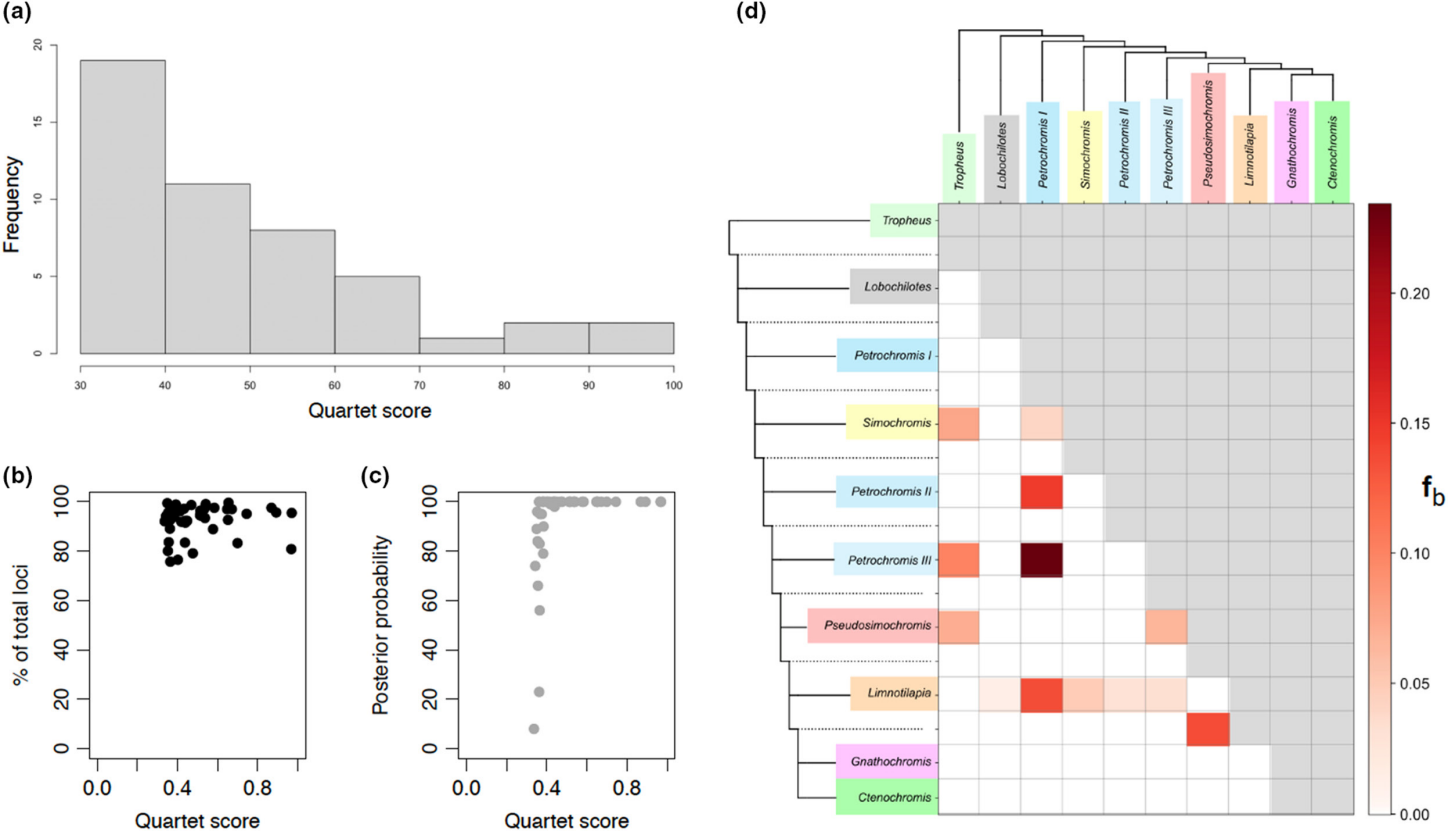
Extent of incomplete lineage sorting (ILS) and hybridization in the Tropheini. (a) Frequency of normalized quartet scores, i.e., proportion of gene tree quartets consistent with a given branch (higher scores indicate lower discordance) (b) relationship of ASTRAL per‐branch quartet scores and the proportion of loci that are informative for its calculation (c) relationship of ASTRAL quartet scores and node posterior probability per branch (d) F‐branch (f_b_) statistics among Tropheini genera

ILS results in incongruence between gene tree topologies (Suh et al., 2015). We calculated quartet scores from ASTRAL that are a measure of gene tree discordance, and this score was used as a proxy for ILS. It must be noted that this discordance can result from others factor too, such as hybridization. The overall normalized quartet score for the 532 loci was 64%. Thus, 64% of gene tree branches were concordant with those in the species tree. The branch‐specific quartet scores ranged from 34 to 97% (Figure 1, and Figures 3a–c). Most quartet scores across the phylogeny were less than 50% and the highest quartet score within the Tropheini was ~65% in *Petrochromis* clade III (the *P. famula* clade). Branches with quartet scores >40% still had high posterior probabilities (Figure 3c).

We investigated excess derived allele sharing as an indicator of gene flow using F‐branch (f_b_) statistics along the Tropheini phylogeny (Figure 3d). F‐branch summarizes many F4 admixture ratio tests (a type of ABBA/BABA statistic, see File S3; Patterson et al., 2012) and thus can identify patterns of allele sharing between non‐sister branches of a phylogeny that are consistent with gene flow (Malinsky et al., 2020). The strongest evidence of excess allele sharing was observed between *Petrochromis* clades I and III (f_b_ = 0.23). There was also evidence of allele sharing between *Petrochromis* clade I and II (f_b_ = 0.15), *Tropheus* and *Pseudosimochromis* (f_b_ = 0.07), *Tropheus* and *Petrochromis* III (f_b_ = 0.10), *Simochromis* and *Petrochromis* I (f_b_ = 0.04), *Pseudosimochromis* and *Petrochromis* III (f_b_ = 0.07). We also found evidence of excess allele sharing between the ancestor of the carnivorous *Ctenochromis* and *Gnathochromis* and the herbivore *Pseudosimochromis* (f_b_ = 0.14). *Limnotilapia dardennii*, the only monotypic omnivore in the Tropheini, showed evidence of gene flow with *Petrochromis* clade I (f_b_ = 0.14), *Petrochromis* clade II (f_b_ = 0.03), *Petrochromis* clade III (f_b_ = 0.03), *Simochromis* (f_b_ = 0.05) and *Lobochilotes* (f_b_ = 0.01).

### 3.4 Molecular evolutionary patterns in loci associated with jaw, vision, body color genes

**FIGURE 4:**
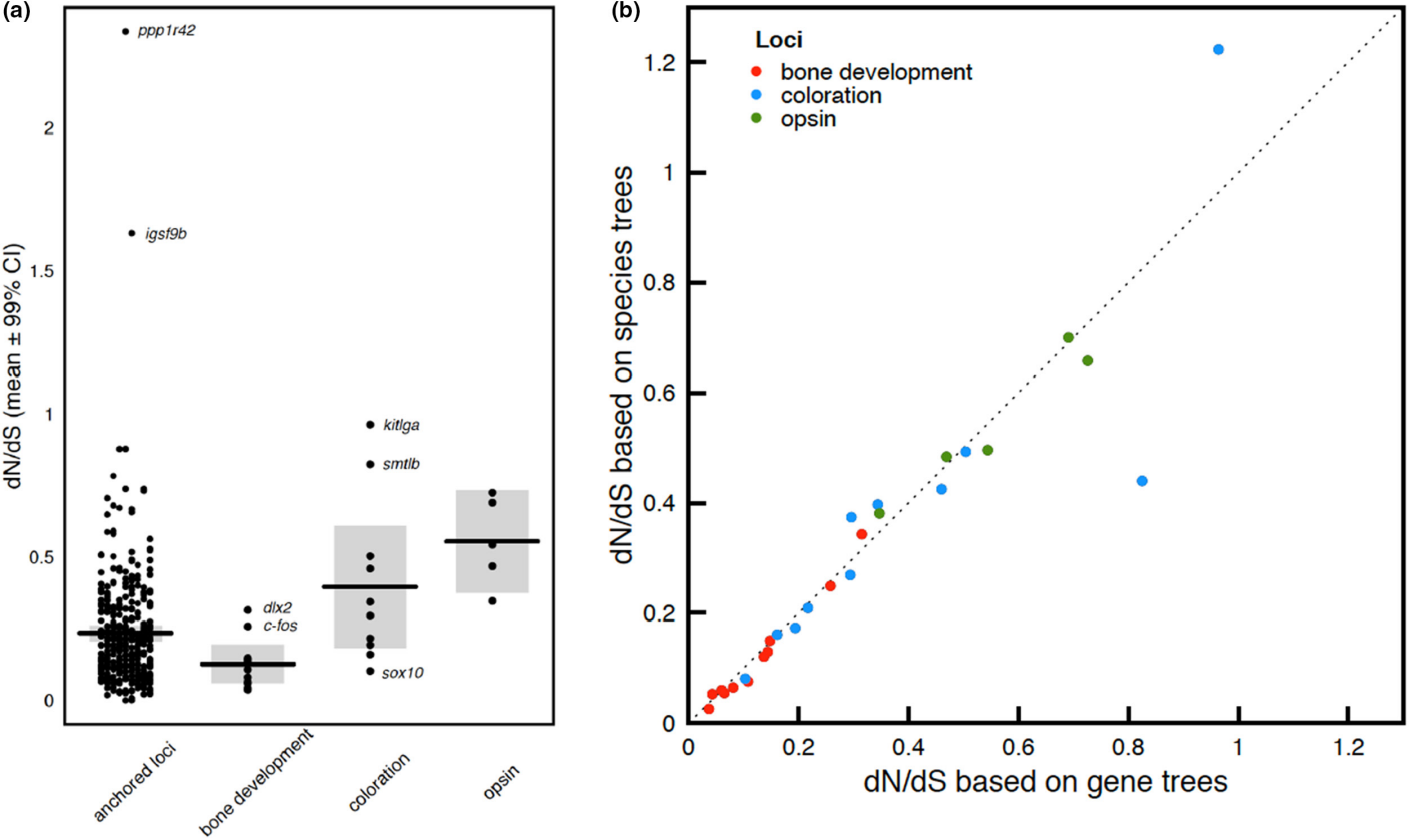
Signatures of selection measured using dN/dS (a) dN/dS across 27 genes implicated in cichlid jaw, vision, and body color adaptations versus all other loci included in the anchored data set (b) concordance of dN/dS calculated using species tree versus gene trees

Three key innovations in Tropheini, and the haplochromines in general, are the presence of (i) highly specialized trophic adaptations (ii) body coloration implicated in sexual selection and (iii) color vision adaptations. Based on published studies, we selected 27 candidate genes associated with these traits to study their molecular evolutionary patterns (File S4a,b). Jaw‐related genes had the lowest coefficients of selection (ω_M0_ or dN/dS) (ω_M0_ = 0.026–0.343, mean ± *SE* = 0.121 ± 0.029; Figure 4a, File S5), and these were on average significantly lower than the background anchored loci (ω_M0_ = 0.0001–2.337, mean ± *SE* = 0.235 ± 0.011; *p* = .003; Figure 4a). Opsin genes showed significantly higher coefficients of selection (ω_M0_ = 0.381–0.700, mean ± *SE* = 0.544 ± 0.059; *p* = .006; Figure 4a) than the loci not considered in any of the three functional categories. Calculation of selection coefficient with gene trees or the species tree did not change the obtained results qualitatively (Figure 4b). There are only two coloration genes that had different dN/dS values between gene and species trees, and this is most likely a reflection of incomplete lineage sorting.

The differences in the coefficients of selection across the three gene classes were also reflected in our results for the analysis of random‐site models of selection. Only two genes in the jaw class (*c‐fos* and *barx1*) showed evidence for variation in selective pressures among codons (M3/M0 random site model test *p* < .05, *X* ^2^‐test with 4 *df*) and none showed evidence of positive selection (detected using M2a/M1a random site model tests, *X* ^2^‐test with 2 *df*; Table S1). In the coloration and opsin genes classes, most of the selected functional loci showed evidence for variation in selective pressures among codons (7 out of 11 and 4 out of 5, respectively, see Table S1) and most showed significant evidence of positive selection (5 out of 11 and 4 out of 5, respectively; Table S1).

Tropheini significantly differed in the rate of molecular evolution from other cichlids in the alignment (based on CmC model, Yang, 2007) only for two jaw (*dlx2*, *creb1*), four opsin (*sws1*, *sws2a*, *rh2b*, *lws*), and three body color genes (*dlc*, *hag*, *mitfa*) (CmC Tropheini: Table S2). Only in half of these genes the Tropheini showed faster rates than outgroups. Given that many of the outgroups were haplochromines from Lake Malawi and Victoria that are known for their diversity, the evidence suggests that Tropheini generally experiences similar rates of molecular evolution for “adaptive” genes as the Lake Malawi and Victoria haplochromines. Similarly, we found only weak evidence for divergence in the rate of molecular evolution among lineages with different feeding habits (CmC‐feeding; ancestral state reconstruction in Figure S4a; Table S3). We were particularly interested in testing for divergence between the highly specialized browser and grazer species within the Tropheini tribe that evolved repeatedly, whereas most of the species in the outgroup are carnivores. The same genes (except *rh2b*) that were found to have variable rates of molecular evolution when looking for Tropheini‐specific rates (CmC‐Tropheini), showed evidence of divergence among trophic groups. In all these genes, the larger differences were between generalist and browsers + grazers (in half of them, generalists showed higher dN/dS). This is reflected by the fact that the model considering the partition of Tropheini species into grazers and browsers (CmC‐feeding) did not fit the data significantly better that the model making no such distinction (i.e., CmC‐Tropheini) for any gene (Table S5–S7). Finally, dimorphisms in term of coloration did not explain a significant amount of variation in the rates of molecular evolution within lineages, except for the two genes *sws2a* and *dlx2* (Table S4).

## 4 DISCUSSION

### 4.1 A phylogenomic hypothesis for the Tropheini

Our coalescent and concatenated phylogenies agreed at deeper nodes with some disagreements found at shallower nodes. Unlike concatenation, ASTRAL coalescent phylogeny accounts for ILS, a phenomenon that is expected in rapid radiations. In agreement with previous findings (Koblmüller et al., 2010; Ronco et al., 2021), the monophyly of the algae browsing genus *Tropheus* and polyphyly of the algae grazing genus *Petrochromis* were strongly supported. Despite overall congruence, our phylogeny disagrees with the genome‐wide phylogeny of Ronco et al. (2021) in the placement of the genera *Simochromis*, *Pseudosimochromis*, and *Limnotilapia*. The position of *Limnotilapia* is switched with *Pseudosimochromis* in our phylogeny versus Ronco et al. (2021). Our phylogeny places the omnivore *Limnotilapia* as sister to the carnivore genera *Ctenochromis* and *Gnathochromis* whereas Ronco et al. (2021) recover it as sister group to these two carnivores plus the algae browser *Pseudosimochromis*. Our phylogeny places *Pseudosimochromis* as sister to the monophyletic *Ctenochromis*, *Gnathochromis* and *Limnotilapia*, whereas Ronco et al. (2021) places it as sister to *Ctenochromis* and *Gnathochromis*. The most surprising discordance is the placement of *Simochromis*. The genome‐wide phylogeny places *Simochromis* as monophyletic with *Petrochomis* clade II and our phylogeny places it as monophyletic with *Gnathochromis*, *Ctenochromis*, *Limnotilapia*, *Pseudosimochromis*, and *Petrochromis* clades II and III. It must be noted that these nodes were interpreted as hard polytomies in the first Tropheini phylogeny solely based on mitochondrial DNA markers (Sturmbauer et al., 2003). The authors of the study argued that the initial radiation of the ancestral Tropheini species into newly available rocky habitats, segregated along the long shoreline of Lake Tanganyika, might have happened very rapidly and in line with an almost contemporary onset of geographic separation. Such a scenario of rapid segregation would almost certainly happen too rapidly to allow for complete lineage sorting. Consequently, part of the topologically relevant mutations were argued to relate to ancient ILS as described by (Takahashi et al., 2001). The first multilocus‐based phylogeny of Koblmüller et al. (2010) supported this idea, as does our ILS analysis as discussed below. The genome‐based phylogeny of Ronco et al. (2021), and now our phylogeny based on ~500 conserved markers show inconsistencies exactly at those initial splits at the base of the radiation. We conclude that not even the vast increase in data led to a clearer phylogenetic signal in these conflicting branches so that the initial hypothesis of Sturmbauer et al. (2003) for explosive speciation via synchronization of genetic divergence, triggered by rapid environmental changes, remains valid. Hence, we suggest that differences in the branching order at the very base of the radiation arise from marker‐specific properties of the data sets. These include mode of inheritance for mitochondrial vs. nuclear markers, chromosome‐ or linkage‐group effects, the intensity and patterning of selected loci, overall conservation, and stochastic errors.

Consistent with previous studies (Koblmüller et al., 2010; Ronco et al., 2021), we found *Petrochromis* to consist of three polyphyletic lineages: Clade I comprised *P. polyodon*‐like species with downward pointing mouths; clade II comprises the upward pointing and terminal mouths of *Interochromis* and *Petrochromis* species; and clade III consists of *P. famula* that have slightly downwards pointing mouths. The ancestral state reconstruction of feeding modes suggests that algae browsing evolved once from the generalist ancestor and switched to algae grazing independently in the three *Petrochromis* clades. However, since we found evidence of gene flow between all three *Petrochromis* clades, we cannot rule out that algae grazing evolved once and then was transferred via adaptive introgression. The ancestral state analysis also suggests that the three specialized modes of carnivory manifested in *L. labiatus*, *C. horei* and *G. pfefferi*, evolved at least in part secondarily from browser ancestors (Figure 2). We do acknowledge that the patterns of trophic specialization evolution purported here are dependent on the phylogenetic hypothesis that we used.

### 4.2 Timing of Tropheini diversification

The Tropheini are considered a model for synchronized speciation with vicariant events likely produced by extreme water level changes (Sturmbauer et al., 2003; Sturmbauer & Meyer, 1992). The most realistic inferred age for the onset of the Tropheini radiation is ~3.8 Ma (95% confidence interval 1.0–8.3 Ma) from our secondary calibration scheme S03, which used fossil‐only calibrations but including upper bound for vicariance node. This estimate for the age of the Tropheini is ~1.0 Ma older than the estimates from Koblmüller et al. (2010) and ~ 0.6 Ma younger than the estimates of Ronco et al. (2021), but confidence intervals are largely overlapping. The timing of the Tropheini coincides with an expansion of the proto‐LT ~3.6 Ma (Cohen et al., 1997), perhaps linking their success to the expanding littoral habitats and shorelines (Cohen et al., 1997; McGlue et al., 2008), which allowed them to radiate while competing with other ongoing radiations. Lake‐level fluctuations act as a “species‐pump” (Rossiter, 1995; Sefc, Mattersdorfer, Ziegelbecker, et al., 2017; Sturmbauer, 1998) promoting allopatric speciation and secondary contact, and it seems plausible that isolation pockets along the stretches of rocky shore (about 1000 km shoreline) of LT promoted the repeated evolution of trophic ecologies, such as algae grazing in the *Petrochromis* complex in different rocky shore sections of the lake. Given that about 120 distinct populations or sister species exist in the genus *Tropheus* alone (Konings, 2019), one must conclude that novel entities evolved and continue to evolve in rocky shorelines that are isolated from each other. According to the species pump hypothesis, such novel entities first have a highly restricted distribution, but gradually spread in the course of lake level fluctuations (Rossiter, 1995; Sturmbauer, 1998).

### 4.3 Extent of incomplete lineage sorting and gene flow

ILS is pervasive in rapidly radiating lineages. In line with previous studies (Koblmüller et al., 2010; Meyer et al., 2017; Svardal et al., 2021), we found substantial discordance between gene trees and the species tree for the Tropheini with only 64% agreement of gene tree quartets with the species tree. In cases where this discordance was not caused by gene flow, it was likely an indicator of ILS, along the lines described in Takahashi et al. (2001) and discussed above. The extent of ILS in the Tropheini was similar to the levels found in the much younger Lake Malawi and Victoria species included in this dataset. As our dataset mostly contains protein‐coding genes that are subjected to purifying selection, we might be underestimating the genome‐wide ILS, as it will likely be higher in neutrally evolving regions (Pease & Hahn, 2013). Another key feature of cichlid radiations is ancient hybridization. Hybridization is thought to play a large role in promoting adaptation and speciation in cichlids, particularly at the onset of a radiation (Genner & Turner, 2012; Irisarri et al., 2018; Meier et al., 2017). We did not find evidence of phylogenetic discordance at the base of the Tropheini radiation that could suggest basal hybridization, as previously reported (Ronco et al., 2021). It is likely that lineages not included in our dataset are responsible for such gene flow signal. Overall, there was some evidence of intergeneric hybridization in Tropheini. This was expected as lake‐level fluctuations are expected to promote secondary contact of spatially separated populations (Sefc, Mattersdorfer, Ziegelbecker, et al., 2017; Sturmbauer, 1998). The evidence of gene flow that we found was (i) between the three algae grazing *Petrochromis* clades and (ii) between the algae browsing species: *Simochromis*, *Pseudosimochromis* and *Tropheus*. Thus, the repeated and rapid evolution of algae grazing and browsing may have been facilitated by gene flow during lake‐level fluctuations. There was no evidence for gene flow between carnivorous and herbivorous genera, or within the carnivores. This illustrates that trophic specialization results in habitat specificity and reproductive isolation, which leads to speciation. Interestingly, *L. darnennii*, which is an omnivore, shared excess alleles with all the herbivorous genera as well as the carnivore *L. labiatus*.

The concatenated and coalescent phylogenies were mostly concordant, despite widespread ILS and instances of hybridization discussed above. One instance of discordance between the phylogenies was in agreement with the presumed hybrid speciation of the *Tropheus* sp. “Mpimbwe” (Van Steenberge et al., 2018), which illustrates that our anchored multilocus sequencing approach with gene incongruence analyses is a powerful tool to identify hybrids. Another instance of discordance was between *P. orthognathus* and *I. loocki* within the *Petrochromis* clade II (<1.5 Ma), which are all algae grazers that live along the rocky shores of LT. *P. orthognathus* and *I. loocki* are more similar morphologically, with terminal mouths, while *P. fasciolatus* has an upward pointing mouth and all other *Petrochromis* have downward pointing mouths (Konings, 2019; Wanek & Sturmbauer, 2015).

### 4.4 Molecular evolution of vision, color, and jaw‐associated genes in the Tropheini

We studied substitution rates (dN/dS) in relation to gene function, phylogeny (lineage effects), ecology, and life‐history traits. Our average background dN/dS of 0.23 was similar to the genome‐wide dN/dS value of 0.20 generated for the Tropheini by Ronco et al. (2021) using whole‐genome sequencing data. Overall, we observed low sequence variation in the coding sequences, suggesting small degrees of freedom for further change of coding sequences. Our molecular evolutionary analysis of genes previously associated with jaw development found little evidence of positive selection, except for *barx1* and *c‐fos* genes; and *dlx2a* that had the highest dN/dS ratio compared to other jaw genes. *Dlx2a* and *c‐fos* regulate calcium dependent effects on jaw development and *dlx* genes are involved in the dorsoventral patterning of pharyngeal arches (reviewed in [Ahi, 2016]). *Barx1* is part of the Notch pathway that regulates facial cartilage and bone formation in zebrafish (Barske et al., 2016). It must be noted that all selected jaw genes are significantly differentially expressed in the jaw apparatus of Tropheini species (Figure S5; data from Singh et al., 2017). This suggests that regulatory variation may be playing a more important role in the evolution of divergent jaws in the Tropheini than coding variation (Ahi et al., 2019; Singh et al., 2017, 2021), and perhaps even in their convergence.

Opsin evolution is linked to habitat adaptation, sexual selection, and food detection in cichlids (O'Quin et al., 2010; Schneider et al., 2020; Sugawara et al., 2005) and these genes had the highest selection coefficients in the Tropheini. The best‐fit CmC model suggests opsin proteins may have evolved in response to selection pressures associated with feeding. The evolution of sexual dichromatism, or increased social complexity, may point to intensified sexual selection in Tropheini species. The molecular evolution of only one opsin gene (*sws2a*) was significantly associated with divergence between sexual monochromatism dichromatism in this clade. *Sws2a* has sex‐specific expression in cichlids (Wright et al., 2020). *Kitlga*, a body color gene involved in melanocyte formation (Hultman et al., 2007) had the highest dN/dS ratio of the coloration genes and also showed evidence of positive selection. Interestingly, the *hagoromo* gene, which has been implicated in the generation of color variation and consequently also speciation in haplochromine cichlids (Terai et al., 2003), was under positive selection in the Tropheini. Overall, purifying selection was prevalent in almost all the loci in this study, including those associated with key adaptations of the Tropheini, except for the *ppp1r42* and *igsf9b* genes that exhibit strong signatures of positive selection. *ppp1r42* is involved in regulating activity of protein phosphatase complexes in the testes during spermatogenesis (Wang & Sperry, 2008) and *igsf9b* regulates inhibitory synapse development in interneurons. Interestingly, this gene is associated with intelligence and behavior in humans (Davies et al., 2018).

## 5 CONCLUSION

We present a robust phylogenetic hypothesis for the Tropheini radiation, a model for studying the evolution of herbivory and carnivory at short timeframes. Based on our results, we propose that the high degree of trophic specialization and trophic transitions in the Tropheini are a result of periods of isolation where populations were restricted by habitat barriers along the lake shore, followed by secondary contact and gene flow between divergent populations resulting from fluctuating lake levels. Instances of gene flow likely facilitated the repeated evolution of algae grazing and algae browsing in the Tropheini. Furthermore, in addition to jaws, vision in Tropheini species likely evolved in response to selection pressures associated with trophic ecology. We conclude that the Tropheini are an important model to study ecological speciation resulting from highly specialized trophic adaptations.

## AUTHOR CONTRIBUTIONS

**Pooja Singh:** Formal analysis (lead); investigation (lead); project administration (lead); writing – original draft (lead); writing – review and editing (lead). **Iker Irisarri:** Formal analysis (supporting); writing – review and editing (supporting). **Julian Torres‐Dowdall:** Formal analysis (supporting); writing – review and editing (supporting). **Gerhard G Thallinger:** Formal analysis (supporting); writing – review and editing (supporting). **Hannes Svardal:** Formal analysis (supporting); writing – review and editing (supporting). **Emily Claire Moriarty Lemmon:** Methodology (lead). **Alan R. Lemmon:** Methodology (lead). **Stephan Koblmüller:** Investigation (supporting); writing – review and editing (supporting). **Axel Meyer:** Conceptualization (equal); funding acquisition (equal); writing – review and editing (supporting). **Christian Sturmbauer:** Conceptualization (equal); funding acquisition (equal); writing – review and editing (supporting).

## CONFLICT OF INTEREST

There is no conflict of interest to declare.

### OPEN RESEARCH BADGES

This article has earned an Open Data badge for making publicly available the digitally‐shareable data necessary to reproduce the reported results. The data is available at https://github.com/poojasingh09/2022_Singh_et_al_Tropheini_phylogenomics.

## Supporting information


Figure S1–S5

Table S1–S7

File S1

File S2

File S3

File S4

File S5

## Data Availability

DNA sequence alignment is available on Dryad: https://doi.org/10.5061/dryad.jh9w0vtcx.
