# Supplementary material for: Phylogenomics of trophically diverse cichlids disentangles processes driving adaptive radiation and repeated trophic transitions
Source: Ecol Evol. 2022 Jul 17;12(7):e9077. doi: 10.1002/ece3.9077 (PMC9288888; doi:10.1002/ece3.9077)
Supplement: Supplementary file 1 — Figure S1–S5 [file ECE3-12-e9077-s001.docx]

## 12. Supplementary Figures


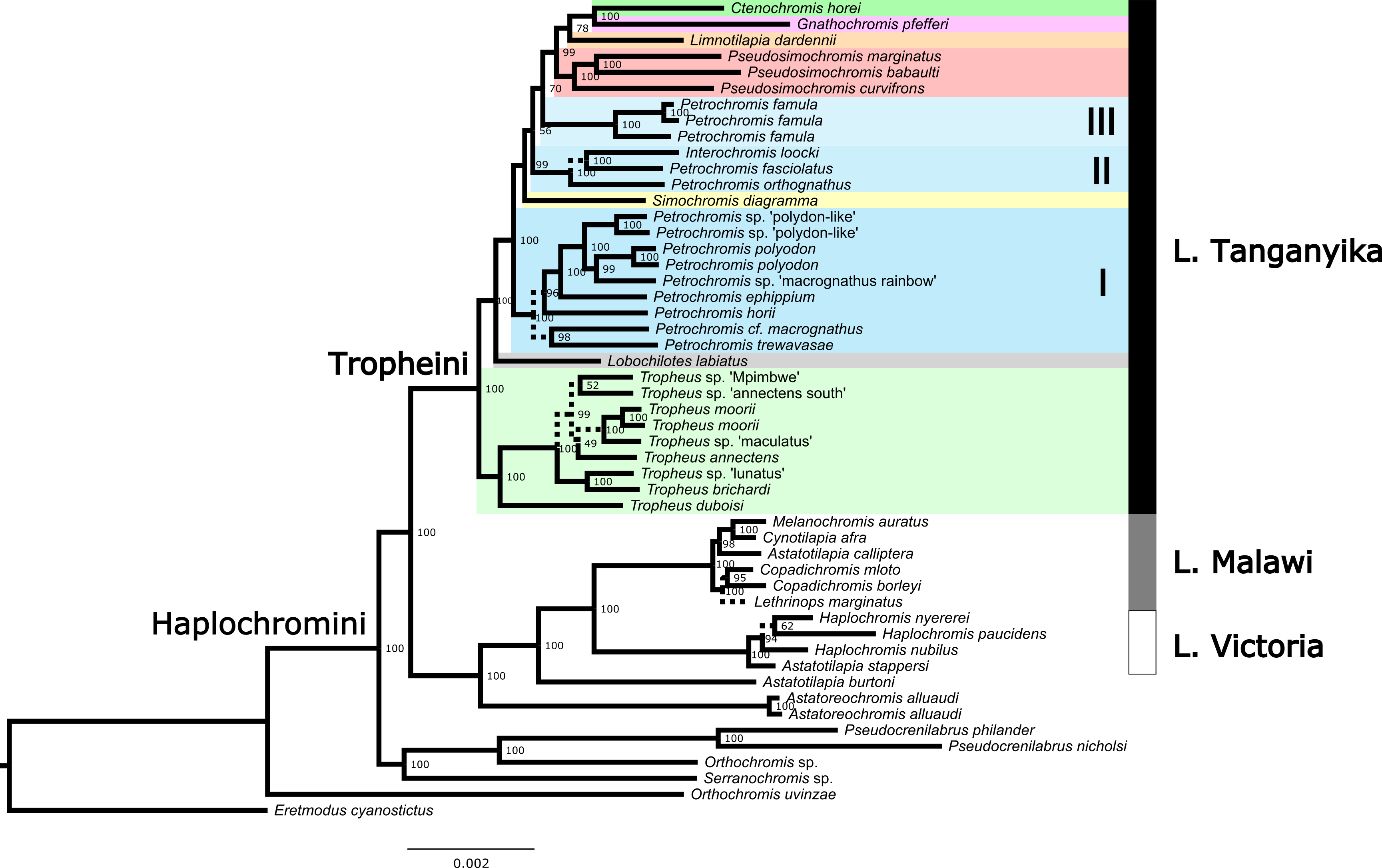


### Supplementary Figure 1 RAxML phylogeny of the Tropheini. Numbers at nodes are bootstrap values. Broken branches denote topology discordance between the RAxML and Astral coalescent phylogenies.


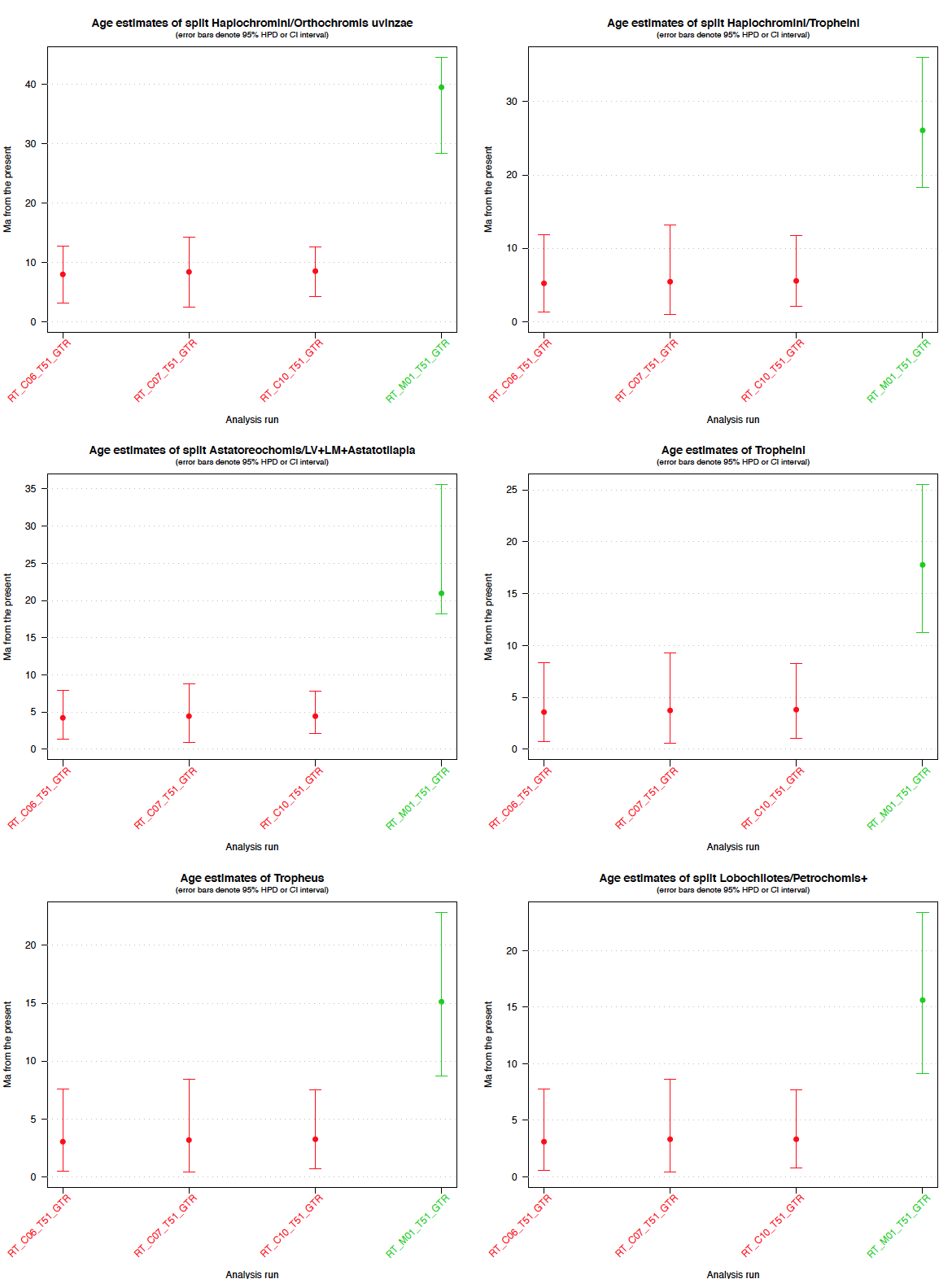


### Supplementary Figure 2 Variance in age estimates for splits in the Tropheini phylogeny using four calibration schemes.


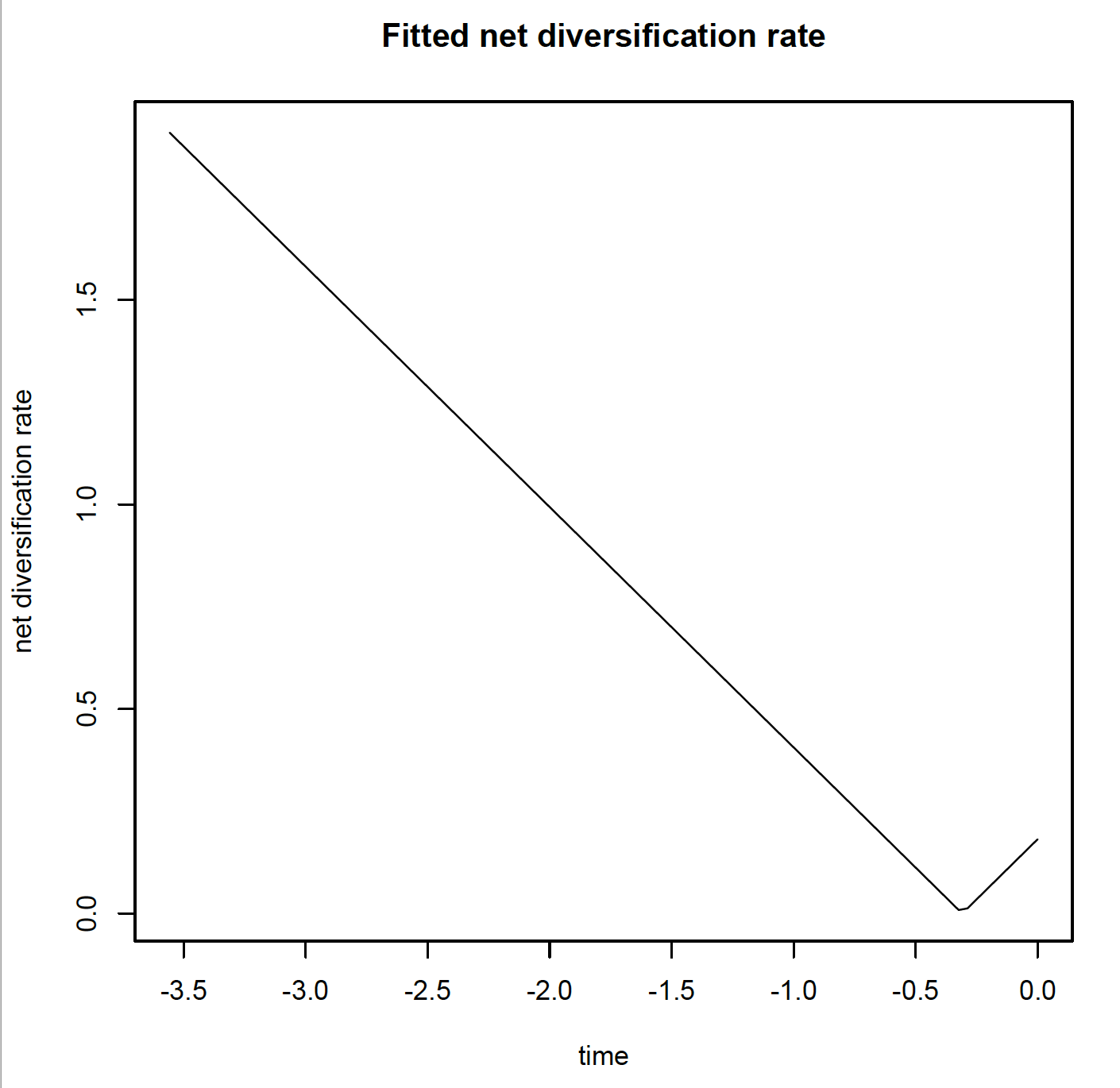


### Supplementary Figure 3 Fitted net diversification rates during Tropheini diversification.


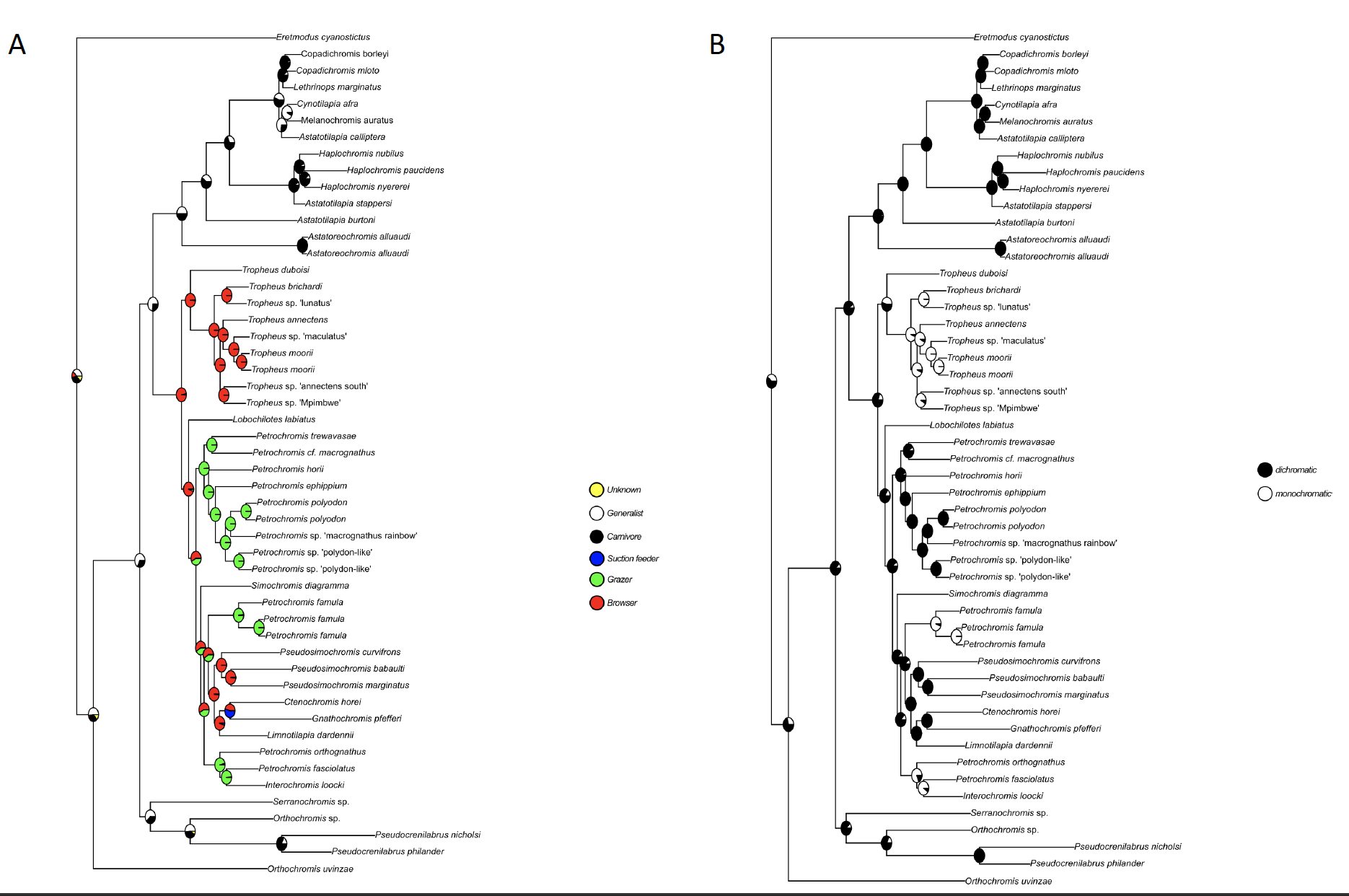


### Supplementary Figure 4 Ancestral state reconstruction of Tropheini (A) feeding modes and (B) sexual dimorphism.

### Supplementary Figure 5 Differential expression of candidate jaw associated genes included in the anchored loci between the jaws of Tropheini species. Red blocks denote significant comparisons between species and the p value after correcting for multiple testing is included in the blocks. Data from Singh et al 2017.

## 13. Supplementary Tables and Files

### Supplementary Table 1. Likelihood ratio test of positive selection (Random sites model in PAML) for the coding sequence of 27 genes captured with targeted enrichment.

### Supplementary Table 2. Clade model C analyses in PAML testing the null hypothesis that there is no divergence in the rate of molecular evolution between Tropheini and other cichlids in our phylogeny (CmC-Tropheini).

### Supplementary Table 3. Clade model C analyses in PAML testing the null hypothesis that there is no divergence in the rate of molecular evolution among cichlid species with different feeding modes (CmC-Feeding).

### Supplementary Table 4. Clade model C analyses in PAML testing the null hypothesis that there is no divergence in the rate of molecular evolution between sexually monochromatic and sexually dichromatic cichlid species (CmC-dimorphism).

### Supplementary Tables 5, 6, 7. Model fitting for analyses of molecular evolution.

### Supplementary File 1 Species and sampling information.

### Supplementary File 2 RelTime divergence time estimates for S01 – S04 secondary calibration schemes.

### Supplementary File 3 D-statistics results from Dsuite.

### Supplementary File 4A Functional genes included in the anchored loci.

### Supplementary File 4B Anchored loci to functional gene correspondence.

### Supplementary File 5 dN/dS values of all anchored sequencing loci.
