# Supplementary material for: Phylogenomics of trophically diverse cichlids disentangles processes driving adaptive radiation and repeated trophic transitions
Source: Ecol Evol. 2022 Jul 17;12(7):e9077. doi: 10.1002/ece3.9077 (PMC9288888; doi:10.1002/ece3.9077)
Supplement: Supplementary file 2 — Table S1–S7 [file ECE3-12-e9077-s002.docx]

# Singh et al Supplementary Tables

## Supplementary Table 1. Likelihood ratio test of positive selection (Random sites model in PAML) for the coding sequence of 27 genes captured with targeted enrichment.

|  |  |  |  |  |  | **Loglikelihood ratio tests^b^** | |  | **Parameters under M2a** | | **Positively selected sites**  **(M2a, BEB)^c^** |  |
| --- | --- | --- | --- | --- | --- | --- | --- | --- | --- | --- | --- | --- |
| **Gene** | **s^a^** | **p_US_^a^** | **ls^a^** | **p_CDS_^a^** | **ω_M0_** | **M3/M0** | **M2a/M1a** |  | **ω_1_** | **ω_3_** |  |  |
| ***genes involved in coloration*** | | | | | | |  |  |  |  |  |  |
| *csf1ra* | 51 | (1.0) | 984 | (1.0) | 0.209 | **60.38*** | **8.94***** |  | 0.107 (0.966) | 3.229 (0.034) | 99, 100 |  |
| *dlc* | 51 | (1.0) | 683 | (0.9) | 0.397 | **451.32*** | **188.76*** |  | 0.058 (0.895) | 12.830 (0.036) | 94, 114, 403, 489, 704, 708, 709, 714, 720, 721 |  |
| *fbxo36b* | 51 | (0.8) | 290 | (1.0) | 0.493 | 10.06^NS^ | 3.25^NS^ |  | 0.246 (0.940) | 4.300 (0.060) |  |  |
| *hag* | 51 | (0.9) | 390 | (1.0) | 0.374 | **137.89*** | **50.48*** |  | 0.017 (0.862) | 8.849 (0.028) |  |  |
| *kir7.1* | 50 | (0.1) | 173 | (0.5) | 0.161 | 0^NS^ | 0^NS^ |  | 0.161 (1) | 0 (0) |  |  |
| *kir7.2* | 40 | (0.8) | 173 | (0.5) | 0.425 | **20.03*** | 7.07^NS^ |  | 0.120 (0.930) | 5.177 (0.070) |  |  |
| *kita* | 51 | (1.0) | 987 | (1.0) | 0.269 | **98.44*** | **23.31*** |  | 0.011 (0.812) | 9.880 (0.015) | 32, 46, 62, 64, 117, 185, 193, 195, 394, 745 |  |
| *kitlga* | 51 | (0.6) | 265 | (0.9) | 1.223 | **45.69*** | **38.89*** |  | 0 (0.256) | 60.268 (0.012) | 40, 82, 83, 109, 170, 171, 172, 192, 193 |  |
| *mitfa* | 51 | (0.9) | 402 | (1.0) | 0.173 | **14.47*** | 0.21^NS^ |  | 0.073 (0.966) | 3.059 (0.034) |  |  |
| *smtlb* | 51 | (0.6) | 230 | (1.0) | 0.440 | 5.11^NS^ | 5.80^NS^ |  | 0.215 (0.967) | 7.710 (0.033) |  |  |
| *sox10* | 51 | (0.9) | 464 | (1.0) | 0.081 | 9.85^NS^ | 0.99^NS^ |  | 0.039 (0.972) | 1.655 (0.028) |  |  |
| ***genes involved in vision*** | | | | | | |  |  |  |  |  |  |
| *sws1* | 51 | (1.0) | 335 | (1.0) | 0.484 | **35.26*** | **8.92*** |  | 0 (0.620) | 8.251 (0.014) | **114***, 204* |  |
| *sws2b* | 51 | (1.0) | 352 | (1.0) | 0.700 | **81.08*** | **41.45*** |  | 0 (0.658) | 11.240 (0.035) | **5**, **45**, **79**, **105**, **234**, **269*** |  |
| *sws2a* | 51 | (0.9) | 352 | (1.0) | 0.381 | **49.79*** | **24.22*** |  | 0 (0.766) | 14.157 (0.018) | **46**, 117*, 248, 287, **335** |  |
| *rh2b* | 51 | (0.9) | 256 | (0.7) | 0.496 | 4.31^NS^ | 1.08^NS^ |  | 0 (0.745) | 1.993 (0.255) |  |  |
| *lws* | 51 | (0.8) | 358 | (1.0) | 0.658 | **113.85*** | **49.45*** |  | 0 (0.826) | 8.053 (0.066) | 7, **10**, **164***, **166**, 203*, 209, **214**, 248, 261*, 262*, **304** |  |
| ***genes involved in jaw development*** | | | | | | | | | | |  | |
| *col6a1* | 51 | (0.9) | 140 | (0.1) | 0.130 | 6.81^NS^ | 4.99^NS^ |  | 0 (0.924) | 2.063 (0.076) |  |  |
| *c-fos* | 51 | (0.9) | 378 | (1.0) | 0.249 | **18.05*** | 2.61^NS^ |  | 0.098 (0.952) | 3.290 (0.048) | 106 |  |
| *creb1* | 51 | (0.7) | 319 | (1.0) | 0.055 | 0^NS^ | 0^NS^ |  | 0.055 (1) | 0 (0) |  |  |
| *barx1* | 51 | (0.6) | 244 | (1.0) | 0.076 | **16.81*** | 5.93^NS^ |  | 0.033 (0.994) | 15.672 (0.006) |  |  |
| *pitx2* | 51 | (0.2) | 68 | (0.3) | 0.014 | 6.58 ^NS^ | 0.75^NS^ |  | 0 (0.980) | 2.897 (0.020) |  |  |
| *runx2b* | 51 | (0.7) | 304 | (0.7) | 0.018 | 0^NS^ | 0^NS^ |  | 0.026 (1) | 0 (0) |  |  |
| *shh* | 51 | (0.8) | 416 | (1.0) | 0.150 | 2.44^NS^ | 0.75^NS^ |  | 0.132 (0.997) | 14.242 (0.003) |  |  |
| *bmp2* | 51 | (0.2) | 119 | (0.3) | 0.060 | 0^NS^ | 0^NS^ |  | 0.060 (1) | 0 (0) |  |  |
| *dlx2* | 51 | (0.5) | 276 | (1.0) | 0.343 | 10.78^NS^ | 4.16^NS^ |  | 0 (0.919) | 4.448 (0.081) |  |  |
| *bmp4* | 51 | (0.3) | 120 | (0.3) | 0.121 | 0^NS^ | 0^NS^ |  | 0.121 (1) | 0 (0) |  |  |
| *sp7* | 51 | (0.6) | 461 | (1.0) | 0.065 | 5.16^NS^ | 0.43^NS^ |  | 0.031 (0.993) | 5.203 (0.007) |  |  |

^a^ **s**: number of sequences; **p_US_**: proportion of unique sequences; **ls**: length of sequences; **p_CDS_**: proportion of the CDS captured.

^b^ **P* < 0.05; ***P* < 0.01; ****P* < 0.001. ^NS^ *P* > 0.05

^c^ Only sites with a posterior probability higher than 85% are reported. If the posterior probability of a site belonging to the positively selected class (ω_2_) is 0.90>*P*>0.95 the site number is underlined and if it is ***P*<0.95** it is in bold. Genes involved in vision (opsin genes) sites are numbered following bovine rhodopsin. Sites under selection in the opsin genes that are located in the binding pocket of the chromophore are indicated with (*)

## Supplementary Table 2. Clade model C analyses in PAML testing the null hypothesis that there is no divergence in the rate of molecular evolution between Tropheini and other cichlids in our phylogeny (CmC-Tropheini). The analyses were run for a subset of 27 genes associated with jaw development, coloration, and colour vision. For each gene, we used a likelihood ratio test (LRT) to determine if a model including partition taxa by phylogeny (i.e., Tropheini versus outgroups) fitted the data significantly better than a null model having no such partition (M2a-rel; Weadick and Chang 2010). The significance (*) of LRT was determined after correcting for multiple testing (FDR=0.05). Values of the coefficient of selection are shown for the two assumed taxa partitions (background/Tropheini). Analyses performed with PAML clade C models, which assume three site classes: class 0 (negatively selected), class 1 (neutral sites; not shown) and class 2 (variable). *p* indicates the proportion of codons in each site class.

|  | LRT |  | Site class 0 | |  | Site class 2 | | | | |
| --- | --- | --- | --- | --- | --- | --- | --- | --- | --- | --- |
| Gene | 2∆*ln*L |  | ω | *p* |  | Background ω | Tropheini ω | | *p* |  |
| *Jaw development genes* | | | | | | | | | | |
| ***dlx2*** | **12.705*** |  | **0.123** | **(0.970)** |  | **0** | **50.691** | **(0.030)** | | |
| ***creb1*** | **10.132*** |  | **0** | **(0)** |  | **0.171** | **0** | **(1)** | | |
| *barx1* | 3.852^NS^ |  | 0.025 | (0.991) |  | 22.384 | 0 | (0.009) | | |
| *runx2b* | 3.453^NS^ |  | 0 | (0) |  | 0.066 | 0 | (1) | | |
| *col6a1* | 2.402^NS^ |  | 0.047 | (0.969) |  | 6.762 | 0 | (0.031) | | |
| *sp7* | 2.244^NS^ |  | 0 | (0.057) |  | 0.074 | 0 | (0.979) | | |
| *pitx2* | 1.921^NS^ |  | 0.027 | (0.995) |  | 0 | 17.795 | (0.005) | | |
| *shh* | 1.336^NS^ |  | 0.129 | (0.996) |  | 15.909 | 0 | (0.004) | | |
| *c-fos* | 0.198^NS^ |  | 0.043 | (0.857) |  | 2.898 | 6.866 | (0.018) | | |
| *bmp2* | 0^NS^ |  | 0.060 | (1) |  | – | – | (0) | | |
| *bmp4* | 0^NS^ |  | 0.121 | (1) |  | – | – | (0) | | |
| *Coloration genes* | | | | | | | | | | |
| ***dlc*** | **32.881*** |  | **0.034** | **(0.857)** |  | **3.764** | **26.351** | **(0.030)** | | |
| ***hag*** | **13.821*** |  | **0** | **(0.832)** |  | **2.384** | **29.366** | **(0.013)** | | |
| ***mitfa*** | **7.977*** |  | **0.480** | **(0.946)** |  | **3.739** | **0** | **(0.054)** | | |
| ***kir7.2*** | **6.179*** |  | **0.127** | **(0.936)** |  | **0.948** | **9.643** | **(0.064)** | | |
| *smtlb* | 3.992^NS^ |  | 0.180 | (0.955) |  | 19.747 | 3.104 | (0.045) | | |
| *sox10* | 3.576^NS^ |  | 0 | (0) |  | 0.063 | 0 | (0.947) | | |
| *kita* | 2.172^NS^ |  | 0.091 | (0.945) |  | 7.015 | 2.601 | (0.045) | | |
| *fbxo36b* | 1.519^NS^ |  | 0.146 | (0.751) |  | 3.940 | 9.813 | (0.025) | | |
| *kitlga* | 0.999^NS^ |  | 0.787 | (0.987) |  | 40.184 | 99.478 | (0.012) | | |
| *kir7.1* | 0.678^NS^ |  | 0 | (0.002) |  | 0.242 | 0 | (0.998) | | |
| *csf1ra* | 0.310^NS^ |  | 0.104 | (0.964) |  | 3.487 | 2.781 | (0.036) | | |
| *Color vision genes* | | | | | | | | | | |
| ***sws2a*** | **28.074*** |  | **0** | **(0.744)** |  | **0** | **133.041** | **(0.008)** | | |
| ***lws*** | **26.900*** |  | **0.038** | **(0.911)** |  | **14.551** | **0.715** | **(0.089)** | | |
| ***sws1*** | **14.163*** |  | **0** | **(0.615)** |  | **17.301** | **0** | **(0.016)** | | |
| ***rh2b*** | **7.935*** |  | **0** | **(0.677)** |  | **10.162** | **0** | **(0.120)** | | |
| *sws2b* | 0.001^NS^ |  | 0 | (0.657) |  | 11.164 | 11.341 | (0.035) | | |

## Supplementary Table 3. Clade model C analyses in PAML testing the null hypothesis that there is no divergence in the rate of molecular evolution among cichlid species with different feeding modes (CmC-Feeding). The analyses were run for a subset of 27 functionally important genes associated with jaw development, coloration, and colour vision. For each gene, we used a likelihood ratio test (LRT) to determine if a model including partition taxa by different feeding modes (i.e., generalists, grazers, browsers) fitted the data significantly better than a null model having no such partition (M2a-rel; Weadick and Chang 2010). The significance (*) of LRT was determined after correcting for multiple testing (FDR=0.05). Values of the coefficient of selection are shown for the three assumed partitions (generalists/grazers/browsers). Analyses performed with PAML clade C models, which assume three site classes: class 0 (negatively selected), class 1 (neutral sites; not shown) and class 2 (variable). *p* indicates the proportion of codons in each site class.

|  | LRT |  | Site class 0 | |  | Site class 2 ^a^ | | | |  |
| --- | --- | --- | --- | --- | --- | --- | --- | --- | --- | --- |
| *Gene* | 2∆*ln*L |  | ω | *p* |  | Generalist ω | Grazer ω | Browser ω | (0.898) | |
| *Jaw development genes* | | | | | | | | | | |
| ***dlx2*** | **14.787*** |  | **0.128** | **(0.971)** |  | **0** | **99.849** | **29.346** | **(0.029)** | |
| ***creb1*** | **10.130*** |  | **0** | **(0)** |  | **0.171** | **0** | **0** | **(1)** | |
| *shh* | 6.597^NS^ |  | 0 | (0.783) |  | 0.868 | 1.492 | 0 | (0.217) | |
| *pitx2* | 6.439^NS^ |  | 0.027 | (0.996) |  | 0 | 77.172 | 0 | (0.004) | |
| *barx1* | 3.852^NS^ |  | 0.025 | (0.991) |  | 22.355 | 0 | 0 | (0.009) | |
| *runx2b* | 3.449^NS^ |  | 0 | (0) |  | 0.066 | 0 | 0 | (1) | |
| *c-fos* | 3.418^NS^ |  | 0 | (0.794) |  | 1.219 | 30.819 | 0 | (0.005) | |
| *sp7* | 2.968^NS^ |  | 0 | (0.967) |  | 2.879 | 0 | 2.377 | (0.033) | |
| *col6a1* | 1.057^NS^ |  | 0 | (0) |  | 0 | 0: | 0.052 | (0.898) | |
| *bmp2* | 0^NS^ |  | 0.060 | (1) |  | – | – | – | (0) | |
| *bmp4* | 0^NS^ |  | 0.121 | (1) |  | – | – | – | (0) | |
| *Coloration genes* | | | | | | | | | | |
| ***dlc*** | **32.882*** |  | **0.035** | **(0.858)** |  | **3.759** | **25.812** | **26.675** | **(0.030)** | |
| ***hag*** | **14.227*** |  | **0** | **(0.832)** |  | **2.355** | **24.673** | **32.662** | **(0.013)** | |
| ***mitfa*** | **10.007*** |  | **0.049** | **(0.947)** |  | **3.762** | **0** | **0** | **(0.053)** | |
| *kir7.2* | 6.898^NS^ |  | 0.130 | (0.937) |  | 0.958 | 17.805 | 7.908 | (0.063) | |
| *sox10* | 5.239^NS^ |  | 0 | (0.789) |  | 0.414 | 0 | 0.410 | (0.194) | |
| *kita* | 4.858^NS^ |  | 0.071 | (0.904) |  | 8.537 | 2.163 | 3.887 | (0.033) | |
| *smtlb* | 4.071^NS^ |  | 0.189 | (0.959) |  | 20.953 | 2.561 | 3.817 | (0.041) | |
| *kitlga* | 1.002^NS^ |  | 0.787 | (0.988) |  | 40.122 | 96.735 | 100.767 | (0.012) | |
| *fbxo36b* | 1.000^NS^ |  | 0.283 | (0.953) |  | 4.168 | 7.720 | 6.551 | (0.040) | |
| *kir7.1* | 0.682^NS^ |  | 0 | (0) |  | 0.242 | 0 | 6.839 | (1) | |
| *csf1ra* | 0.522^NS^ |  | 0.103 | (0.963) |  | 3.484 | 2.392 | 3.063 | (0.037) | |
| *Color vision genes* | | | | | | | | | | |
| ***sws2a*** | **29.766*** |  | **0** | **(0.744)** |  | **0** | **31.430** | **179.864** | **(0.008)** | |
| ***lws*** | **26.716*** |  | **0.040** | **(0.912)** |  | **14.678** | **0** | **1.083** | **(0.088)** | |
| ***sws1*** | **14.163*** |  | **0** | **(0.615)** |  | **3.970** | **0** | **0** | **(0.016)** | |
| *rh2b* | 7.935^NS^ |  | 0 | (0.677) |  | 10.166 | 0 | 0 | (0.120) | |
| *sws2b* | 1.387^NS^ |  | 0 | (0.652) |  | 11.984 | 20.006 | 8.340 | (0.032) | |

## Supplementary Table 4. Clade model C analyses in PAML testing the null hypothesis that there is no divergence in the rate of molecular evolution between sexually monochromatic and sexually dichromatic cichlid species (CmC-dimorphism). The analyses were run for a subset of 27 functionally important genes. For each gene, we used a likelihood ratio test (LRT) to determine if a model including partition taxa by coloration (i.e., monochromatic versus dichromatic species) fitted the data significantly better than a null model having no such partition (M2a-rel; Weadick and Chang 2010). The significance (*) of LRT was determined after correcting for multiple testing (FDR=0.05). Values of the coefficient of selection are shown for the two assumed taxa partitions (monochromatic/dichromatic). Analyses performed with PAML clade C models, which assume three site classes: class 0 (negatively selected), class 1 (neutral sites; not shown) and class 2 (variable). *p* indicates the proportion of codons in each site class.

|  | | LRT |  | Site class 0 | |  | Site class 2 ^a^ | | |
| --- | --- | --- | --- | --- | --- | --- | --- | --- | --- |
| Gene | | 2∆*ln*L |  | ω | *p* |  | monocromatic ω | dichromatic ω | *p* |
| *Jaw development genes* | | | | | | | | |  |
| ***dlx2*** | | **8.796*** |  | **0** | **(0.797)** |  | **999** | **0** | **(0.021)** |
| *col6a1* | | 2.884^NS^ |  | 0 | (0.923) |  | 0 | 2.216 | (0.076) |
| *sp7* | | 2.576^NS^ |  | 0.042 | (0.997) |  | 108.98 | 6.277 | (0.003) |
| *c-fos* | | 1.677^NS^ |  | 0 | (0.795) |  | 47.466 | 4.323 | (0.005) |
| *runx2b* | | 1.054^NS^ |  | 0 | (<0.001) |  | 0 | 0.035 | (0.999) |
| *barx1* | | 0.881^NS^ |  | 0.032 | (0.993) |  | 0 | 17.483 | (0.006) |
| *creb1* | | 0.734^NS^ |  | 0 | (0.002) |  | 0 | 0.060 | (0.998) |
| *pitx2* | | 0.332^NS^ |  | 0 | (0.980) |  | 0 | 3.036 | (0.020) |
| *shh* | | 0.233^NS^ |  | 0.132 | (0.997) |  | 0 | 15.110 | (0.003) |
| *bmp2* | | 0^NS^ |  | 0.060 | (1) |  | – | – | (0) |
| *bmp4* | | 0^NS^ |  | 0.121 | (1) |  | – | – | (0) |
| *Coloration genes* | | | | | | | | |  |
| *sox10* | | 4.729^NS^ |  | 0.054 | (0.989) |  | 13.005 | 1.443 | (0.011) |
| *hag* | | 4.611^NS^ |  | 0.006 | (0.846) |  | 29.757 | 7.531 | (0.026) |
| *fbxo36b* | | 3.962^NS^ |  | 0.295 | (0.964) |  | 26.165 | 4.255 | (0.036) |
| *mitfa* | | 2.631^NS^ |  | 0.067 | (0.963) |  | 0 | 3.372 | (0.037) |
| *kir7.2* | | 1.318^NS^ |  | 0 | (0.750) |  | 13.598 | 4.470 | (0.037) |
| *csf1ra* | | 1.174^NS^ |  | 0.106 | (0.965) |  | 1.861 | 3.450 | (0.035) |
| *kitlga* | | 0.568^NS^ |  | 0 | (0.258) |  | 183.75 | 56.844 | (0.001) |
| *dlc* | | 0.454^NS^ |  | 0.058 | (0.895) |  | 17.065 | 12.486 | (0.036) |
| *kita* | | 0.347^NS^ |  | 0.010 | (0.810) |  | 5.985 | 10.274 | (0.015) |
| *smtlb* | | 0.256^NS^ |  | 0.208 | (0.967) |  | 4.494 | 8.250 | (0.035) |
| *kir7.1* | | 0.001^NS^ |  | 0.161 | (0.999) |  | 2.974 | 0 | (0.001) |
| *Color vision genes* | | | | | | | | |  |
| ***sws2a*** | | **15.265*** |  | **0** | **(0.746)** |  | **999** | **2.820** | **(0.006)** |
| *sws1* | | 4.102^NS^ |  | 0 | (0.622) |  | 0 | 8.305 | (0.049) |
| *rh2b* | | 1.865^NS^ |  | 0 | (0.672) |  | 0 | 2.613 | (0.177) |
| *lws* | | 0.728^NS^ |  | 0 | (0.835) |  | 0 | 7.986 | (0.071) |
| *sws2b* | | 0.375^NS^ |  | 0 | (0.802) |  | 16.758 | 9.549 | (0.049) |

## Supplementary Table 5. Analysis of molecular divergence in jaw development genes. For each gene, four different models are fitted to the data, which partition taxa by phylogeny (CmC-Tropheini), sexual dimorphism in body colour (present/ absent; CmC-dimorphism), diet (browsers/ grazers/ generalist; CmC-feeding), or no partition (null model M2a-rel; Weadick and Chang 2010). The significance (*) of each model is tested against the null model by LRT after correcting for multiple testing (FDR=0.05) and the relative fit of different models compared by AIC. Values of the coefficient of selection are shown for the two assumed taxa partitions (background/foreground) indicating their phenotype. Analyses performed with PAML clade C models, which assume three site classes: class 0 (negatively selected), class 1 (neutral sites; not shown) and class 2 (variable).

|  |  |  |  | LRT |  | Site class 0 | |  | Site class 2 ^a^ | | | | | | | |  |
| --- | --- | --- | --- | --- | --- | --- | --- | --- | --- | --- | --- | --- | --- | --- | --- | --- | --- |
| Opsin gene | Model tested | *ln*L | ∂AIC | 2∆*ln*L |  | ω | *p* |  | Background ω b | | Foreground ω | | | *p* | | | |
| *col6a1* | CmC-dimorphism | -766.289 | 0 | 2.884^NS^ |  | 0 | (0.923) |  | m.c.: | 0 | d.c.: | 2.216 | | (0.076) | | |  |
|  | CmC-Tropheini | -766.530 | 0.482 | 2.402^NS^ |  | 0.047 | (0.969) |  | basal.: | 6.762 | Tropheini: | 0 | | (0.031) | | |  |
|  | M2a-rel | -767.731 | 0.884 |  |  | 0 | (0.849) |  |  | 0 |  |  | | (0.033) | | |  |
|  | CmC-feeding | -767.203 | 3.828 | 1.057^NS^ |  | 0 | (0) |  | omnivorous | 0 | browsers: | 0.052 | | (0.898) | | |  |
|  |  |  |  |  |  |  |  |  |  |  | grazers: | | 0 | |  |  |  |
| *c-fos* | M2a-rel | -2042.319 | 0 |  |  | 0.098 | (0.952) |  |  | 3.290 |  |  | | (0.048) | | |  |
|  | CmC-dimorphism | -2041.481 | 0.323 | 1.677^NS^ |  | 0 | (0.795) |  | m.c.: | 47.466 | d.c.: | 4.323 | | (0.005) | | |  |
|  | CmC-feeding | -2040.610 | 0.582 | 3.418^NS^ |  | 0 | (0.794) |  | omnivorous | 1.219 | browsers: | 0 | | (0.005) | | |  |
|  |  |  |  |  |  |  |  |  |  |  | grazers: | 30.819 | |  | | |  |
|  | CmC-Tropheini | -2042.220 | 1.802 | 0.198^NS^ |  | 0.043 | (0.857) |  | basal.: | 2.898 | Tropheini: | 6.866 | | (0.018) | | |  |
| *creb1* | CmC-Tropheini | -1576.929 | 0 | 10.132* |  | 0 | (0) |  | basal.: | 0.171 | Tropheini: | 0 | | (1) | | |  |
|  | CmC-feeding | -1576.930 | 2.003 | 10.130* |  | 0 | (0) |  | omnivorous | 0.171 | browsers: | 0 | | (1) | | |  |
|  |  |  |  |  |  |  |  |  |  |  | grazers: | 0 | |  | | |  |
|  | M2a-rel | -1581.995 | 8.132 |  |  | 0.055 | (1) |  |  | – |  |  | | (0) | | |  |
|  | CmC-dimorphism | -1581.628 | 9.399 | 0.734^NS^ |  | 0 | (0.002) |  | m.c.: | 0 | d.c.: | 0.060 | | (0.998) | | |  |
| *barx1* | CmC-Tropheini | -1189.535 | 0 | 3.852^NS^ |  | 0.025 | (0.991) |  | basal.: | 22.384 | Tropheini: | 0 | | (0.009) | | |  |
|  | M2a-rel | -1191.461 | 1.852 |  |  | 0.033 | (0.994) |  |  | 15.837 |  |  | | (0.006) | | |  |
|  | CmC-feeding | -1189.535 | 1.999 | 3.852^NS^ |  | 0.025 | (0.991) |  | omnivorous | 22.355 | browsers: | 0 | | (0.009) | | |  |
|  |  |  |  |  |  |  |  |  |  |  | grazers: | 0 | |  | | |  |
|  | CmC-dimorphism | -1191.020 | 2.971 | 0.881^NS^ |  | 0.032 | (0.993) |  | m.c.: | 0 | d.c.: | 17.483 | | (0.006) | | |  |
| *pitx2* | CmC-feeding | -1452.073 | 0 | 6.439^NS^ |  | 0.027 | (0.996) |  | omnivorous | 0 | browsers: | 0 | | (0.004) | | |  |
|  |  |  |  |  |  |  |  |  |  |  | grazers: | | 77.172 | |  |  |  |
|  | M2a-rel | -1455.293 | 2.439 |  |  | 0 | (0.980) |  |  | 2.898 |  |  | | (0.012) | | |  |
|  | CmC-Tropheini | -1454.333 | 2.519 | 1.921^NS^ |  | 0.027 | (0.995) |  | basal.: | 0 | Tropheini: | 17.795 | | (0.005) | | |  |
|  | CmC-dimorphism | -1455.127 | 4.107 | 0.332^NS^ |  | 0 | (0.980) |  | m.c.: | 0 | d.c.: | 3.036 | | (0.020) | | |  |
| *shh* | CmC-feeding | -2061.889 | 0 | 6.597^NS^ |  | 0 | (0.783) |  | omnivorous | 0.868 | browsers: | 0 | | (0.217) | | |  |
|  |  |  |  |  |  |  |  |  |  |  | grazers: | 1.492 | |  | | |  |
|  | M2a-rel | -2065.187 | 2.597 |  |  | 0.132 | (0.997) |  |  | 14.242 |  |  | | (0.003) | | |  |
|  | CmC-Tropheini | -2064.519 | 3.260 | 1.336^NS^ |  | 0.129 | (0.996) |  | basal.: | 15.909 | Tropheini: | 0 | | (0.004) | | |  |
|  | CmC-dimorphism | -2065.071 | 4.364 | 0.233^NS^ |  | 0.132 | (0.997) |  | m.c.: | 0 | d.c.: | 15.110 | | (0.003) | | |  |
| *bmp2* | M2a-rel | -525.151 | 0 |  |  | 0.060 | (1) |  |  | – |  |  | | (0) | | |  |
|  | CmC-Tropheini | -525.151 | 2.000 | 0^NS^ |  | 0.060 | (1) |  | basal.: | – | Tropheini: | – | | (0) | | |  |
|  | CmC-dimorphism | -525.151 | 2.000 | 0^NS^ |  | 0.060 | (1) |  | m.c.: | – | d.c.: | – | | (0) | | |  |
|  | CmC-feeding | -525.151 | 4.000 | 0^NS^ |  | 0.060 | (1) |  | omnivorous | – | browsers: | – | | (0) | | |  |
|  |  |  |  |  |  |  |  |  |  |  | grazers: | – | |  | | |  |
| *dlx2* | CmC-feeding | -1339.599 | 0 | 14.787* |  | 0.128 | (0.971) |  | omnivorous | 0 | browsers: | 29.346 | | (0.029) | | |  |
|  |  |  |  |  |  |  |  |  |  |  | grazers: | 99.849 | |  | | |  |
|  | CmC-Tropheini | -1340.640 | 0.082 | 12.705* |  | 0.123 | (0.970) |  | basal.: | 0 | Tropheini: | 50.691 | | (0.030) | | |  |
|  | CmC-dimorphism | -1342.595 | 3.991 | 8.796 |  | 0 | (0.797) |  | m.c.: | 999 | d.c.: | 0 | | (0.021) | | |  |
|  | M2a-rel | -1346.993 | 10.787 |  |  | 0 | (0.919) |  |  | 4.448 |  |  | | (0.081) | | |  |
| *bmp4* | M2a-rel | -556.090 | 0 |  |  | 0.121 | (1) |  |  | – |  |  | | (0) | | |  |
|  | CmC-Tropheini | -556.090 | 2.000 | 0^NS^ |  | 0.121 | (1) |  | basal.: | – | Tropheini: | – | | (0) | | |  |
|  | CmC-dimorphism | -556.090 | 2.000 | 0^NS^ |  | 0.121 | (1) |  | m.c.: | – | d.c.: | – | | (0) | | |  |
|  | CmC-feeding | -556.090 | 4.000 | 0^NS^ |  | 0.121 | (1) |  | omnivorous | – | browsers: | – | | (0) | | |  |
|  |  |  |  |  |  |  |  |  |  |  | grazers: | | – | |  |  |  |
| *sp7* | CmC-dimorphism | -2206.245 | 0 | 2.576^NS^ |  | 0.042 | (0.997) |  | m.c.: | 108.98 | d.c.: | 6.277 | | (0.003) | | |  |
|  | CmC-Tropheini | -2206.411 | 0.332 | 2.244^NS^ |  | 0 | (0.057) |  | basal.: | 0.074 | Tropheini: | 0 | | (0.979) | | |  |
|  | M2a-rel | -2207.533 | 0.576 |  |  | 0.031 | (0.993) |  |  | 5.203 |  |  | | (0.007) | | |  |
|  | CmC-feeding | -2206.049 | 1.608 | 2.968^NS^ |  | 0 | (0.967) |  | omnivorous | 2.879 | browsers: | 2.377 | | (0.033) | | |  |
|  |  |  |  |  |  |  |  |  |  |  | grazers: | 0 | |  | | |  |
| *runx2b* | CmC-Tropheini | -1419.381 | 0 | 3.453^NS^ |  | 0 | (0) |  | basal.: | 0.066 | Tropheini: | 0 | | (1) | | |  |
|  | M2a-rel | -1421.108 | 1.453 |  |  | 0.026 | (1) |  |  | – |  |  | | (0) | | |  |
|  | CmC-feeding | -1419.383 | 2.004 | 3.449^NS^ |  | 0 | (0) |  | omnivorous | 0.066 | browsers: | 0 | | (1) | | |  |
|  |  |  |  |  |  |  |  |  |  |  | grazers: | | 0 | |  |  |  |
|  | CmC-dimorphism | -1420.581 | 2.399 | 1.054^NS^ |  | 0 | (<0.001) |  | m.c.: | 0 | d.c.: | 0.035 | | (0.999) | | |  |

^a^ Abbreviations refer to sexually monomorphic in coloration (m.c.), sexually dimorphic in coloration (d.c.).

## Supplementary Table 6. Analysis of molecular divergence in coloration genes. For each gene, four different models are fitted to the data, which partition taxa by phylogeny (CmC-Tropheini), sexual dimorphism in body colour (present/ absent; CmC-dimorphism), diet (browsers/ grazers/ generalist; CmC-feeding)or no partition (null model M2a-rel; Weadick and Chang 2010). The significance (*) of each model is tested against the null model by LRT after correcting for multiple testing (FDR=0.05) and the relative fit of different models compared by AIC. Values of the coefficient of selection are shown for the two assumed taxa partitions (background/foreground) indicating their phenotype. Analyses performed with PAML clade C models, which assume three site classes: class 0 (negatively selected), class 1 (neutral sites; not shown) and class 2 (variable).

|  |  |  |  | LRT |  | Site class 0 | |  | Site class 2 ^A^ | | | | | | | |  |
| --- | --- | --- | --- | --- | --- | --- | --- | --- | --- | --- | --- | --- | --- | --- | --- | --- | --- |
| Opsin gene | Model tested | *ln*L | ∂AIC | 2∆*ln*L |  | ω | *p* |  | Background ω b | | Foreground ω | | | *p* | | | |
| csf1ra | M2a-rel | -5781.440 | 0 |  |  | 0.107 | (0.966) |  |  | 3.229 |  |  | | (0.034) | | |  |
|  | CmC-dimorphism | -5780.853 | 0.826 | 1.174^NS^ |  | 0.106 | (0.965) |  | m.c.: | 1.861 | d.c.: | 3.450 | | (0.035) | | |  |
|  | CmC-Tropheini | -5781.285 | 1.690 | 0.310^NS^ |  | 0.104 | (0.964) |  | basal.: | 3.487 | Tropheini: | 2.781 | | (0.036) | | |  |
|  | CmC-feeding | -5781.179 | 3.478 | 0.522^NS^ |  | 0.103 | (0.963) |  | omnivorous | 3.484 | browsers: | 3.063 | | (0.037) | | |  |
|  |  |  |  |  |  |  |  |  |  |  | grazers: | | 2.392 | |  |  |  |
| dlc | CmC-Tropheini | -4136.156 | 0 | 32.881* |  | 0.034 | (0.857) |  | basal.: | 3.764 | Tropheini: | 26.351 | | (0.030) | | |  |
|  | CmC-feeding | -4136.155 | 1.998 | 32.882* |  | 0.035 | (0.858) |  | omnivorous | 3.759 | browsers: | 26.675 | | (0.030) | | |  |
|  |  |  |  |  |  |  |  |  |  |  | grazers: | 25.812 | |  | | |  |
|  | CmC-dimorphism | -4152.596 | 30.881 | 0.454^NS^ |  | 0.058 | (0.895) |  | m.c.: | 17.065 | d.c.: | 12.486 | | (0.036) | | |  |
|  | M2a-rel | -4152.369 | 32.427 |  |  | 0.055 | (0.890) |  |  | 12.830 |  |  | | (0.036) | | |  |
| *fbxo36b* | CmC-dimorphism | -1431.327 | 0 | 3.962^NS^ |  | 0.295 | (0.964) |  | m.c.: | 26.165 | d.c.: | 4.255 | | (0.036) | | |  |
|  | M2a-rel | -1433.960 | 1.962 |  |  | 0.246 | (0.940) |  |  | 4.287 |  |  | | (0.060) | | |  |
|  | CmC-Tropheini | -1432.97 | 2.444 | 1.519^NS^ |  | 0.146 | (0.751) |  | basal.: | 3.940 | Tropheini: | 9.813 | | (0.025) | | |  |
|  | CmC-feeding | -1433.735 | 6.000 | 1.000^NS^ |  | 0.283 | (0.953) |  | omnivorous | 4.168 | browsers: | 6.551 | | (0.040) | | |  |
|  |  |  |  |  |  |  |  |  |  |  | grazers: | 7.720 | |  | | |  |
| *hag* | CmC-Tropheini | -2179.872 | 0 | 13.821* |  | 0 | (0.832) |  | basal.: | 2.384 | Tropheini: | 29.366 | | (0.013) | | |  |
|  | CmC-feeding | -2179.669 | 1.594 | 14.227* |  | 0 | (0.832) |  | omnivorous | 2.355 | browsers: | 32.662 | | (0.013) | | |  |
|  |  |  |  |  |  |  |  |  |  |  | grazers: | 24.673 | |  | | |  |
|  | CmC-dimorphism | -2184.477 | 9.210 | 4.611^NS^ |  | 0.006 | (0.846) |  | m.c.: | 29.757 | d.c.: | 7.531 | | (0.026) | | |  |
|  | M2a-rel | -2186.783 | 11.821 |  |  | 0.017 | (0.863) |  |  | 8.837 |  |  | | (0.028) | | |  |
| *kir7.1* | M2a-rel | -731.994 | 0 |  |  | 0.161 | (1) |  |  | – |  |  | | (0) | | |  |
|  | CmC-Tropheini | -731.655 | 1.322 | 0.678^NS^ |  | 0 | (0.002) |  | basal.: | 0.242 | Tropheini: | 0 | | (0.998) | | |  |
|  | CmC-feeding | -731.653 | 3.318 | 0.682^NS^ |  | 0 | (0) |  | omnivorous | 0.242 | browsers: | 6.839 | | (1) | | |  |
|  |  |  |  |  |  |  |  |  |  |  | grazers: | | 0 | |  |  |  |
|  | CmC-dimorphism | -731.994 | 5.999 | 0.001^NS^ |  | 0.161 | (0.999) |  | m.c.: | 2.974 | d.c.: | 0 | | (0.001) | | |  |
| *kir7.2* | CmC-Tropheini | -1015.570 | 0 | 6.179^NS^ |  | 0.127 | (0.936) |  | basal.: | 0.948 | Tropheini: | 9.643 | | (0.064) | | |  |
|  | CmC-feeding | -1015.211 | 1.281 | 6.898^NS^ |  | 0.130 | (0.937) |  | omnivorous | 0.958 | browsers: | 7.908 | | (0.063) | | |  |
|  |  |  |  |  |  |  |  |  |  |  | grazers: | 17.805 | |  | | |  |
|  | M2a-rel | -1018.660 | 4.179 |  |  | 0.120 | (0.930) |  |  | 5.177 |  |  | | (0.070) | | |  |
|  | CmC-dimorphism | -1018.001 | 4.861 | 1.318^NS^ |  | 0 | (0.750) |  | m.c.: | 13.598 | d.c.: | 4.470 | | (0.037) | | |  |
| *kita* | CmC-feeding | -5403.024 | 0 | 4.858^NS^ |  | 0.071 | (0.904) |  | omnivorous | 8.537 | browsers: | 3.887 | | (0.033) | | |  |
|  |  |  |  |  |  |  |  |  |  |  | grazers: | 2.163 | |  | | |  |
|  | CmC-Tropheini | -5404.367 | 0.686 | 2.172^NS^ |  | 0.091 | (0.945) |  | basal.: | 7.015 | Tropheini: | 2.601 | | (0.045) | | |  |
|  | M2a-rel | -5405.453 | 0.858 |  |  | 0.011 | (0.812) |  |  | 9.882 |  |  | | (0.015) | | |  |
|  | CmC-dimorphism | -5405.279 | 2.511 | 0.347^NS^ |  | 0.010 | (0.810) |  | m.c.: | 5.985 | d.c.: | 10.274 | | (0.015) | | |  |
| *kitlga* | M2a-rel | -1284.841 | 0 |  |  | 0 | (0.256) |  |  | 60.268 |  |  | | (0.012) | | |  |
|  | CmC-Tropheini | -1284.341 | 1.001 | 0.999^NS^ |  | 0.787 | (0.987) |  | basal.: | 40.184 | Tropheini: | 99.478 | | (0.012) | | |  |
|  | CmC-dimorphism | -1284.556 | 1.432 | 0.568^NS^ |  | 0 | (0.258) |  | m.c.: | 183.75 | d.c.: | 56.844 | | (0.001) | | |  |
|  | CmC-feeding | -1284.340 | 2.998 | 1.002^NS^ |  | 0.787 | (0.988) |  | omnivorous | 40.122 | browsers: | 100.767 | | (0.012) | | |  |
|  |  |  |  |  |  |  |  |  |  |  | grazers: | 96.735 | |  | | |  |
| *mitfa* | CmC-feeding | -2137.021 | 0 | 10.007* |  | 0.049 | (0.947) |  | omnivorous | 3.762 | browsers: | 0 | | (0.053) | | |  |
|  |  |  |  |  |  |  |  |  |  |  | grazers: | | 0 | |  |  |  |
|  | CmC-Tropheini | -2138.036 | 0.030 | 7.977* |  | 0.480 | (0.946) |  | basal.: | 3.739 | Tropheini: | 0 | | (0.054) | | |  |
|  | CmC-dimorphism | -2140.709 | 5.376 | 2.631^NS^ |  | 0.067 | (0.963) |  | m.c.: | 0 | d.c.: | 3.372 | | (0.037) | | |  |
|  | M2a-rel | -2142.024 | 6.007 |  |  | 0.073 | (0.966) |  |  | 3.059 |  |  | | (0.034) | | |  |
| *smtlb* | CmC-Tropheini | -1205.947 | 0 | 3.992^NS^ |  | 0.180 | (0.955) |  | basal.: | 19.747 | Tropheini: | 3.104 | | (0.045) | | |  |
|  | CmC-feeding | -1205.907 | 1.921 | 4.071^NS^ |  | 0.189 | (0.959) |  | omnivorous | 20.953 | browsers: | 3.817 | | (0.041) | | |  |
|  |  |  |  |  |  |  |  |  |  |  | grazers: | 2.561 | |  | | |  |
|  | M2a-rel | -1207.943 | 1.992 |  |  | 0.215 | (0.967) |  |  | 7.711 |  |  | | (0.033) | | |  |
|  | CmC-dimorphism | -1207.815 | 3.736 | 0.256^NS^ |  | 0.208 | (0.967) |  | m.c.: | 4.494 | d.c.: | 8.250 | | (0.035) | | |  |
| sox10 | CmC-dimorphism | -2682.275 | 0 | 4.729^NS^ |  | 0.054 | (0.989) |  | m.c.: | 13.005 | d.c.: | 1.443 | | (0.011) | | |  |
|  | CmC-Tropheini | -2682.852 | 1.153 | 3.576^NS^ |  | 0 | (0) |  | basal.: | 0.063 | Tropheini: | 0 | | (0.947) | | |  |
|  | CmC-feeding | -2682.020 | 1.490 | 5.239^NS^ |  | 0 | (0.789) |  | omnivorous | 0.414 | browsers: | 0.410 | | (0.194) | | |  |
|  |  |  |  |  |  |  |  |  |  |  | grazers: | 0 | |  | | |  |
|  | M2a-rel | -2684.640 | 2.729 |  |  | 0.020 | (0.882) |  |  | 0.020 |  |  | | (0.055) | | |  |

^a^ Abbreviations refer to sexually monomorphic in coloration (m.c.), sexually dimorphic in coloration (d.c.).

## Supplementary Table 7 Analysis of molecular divergence in vision genes. For each gene, four different models are fitted to the data, which partition taxa by phylogeny (CmC-Tropheini), sexual dimorphism in body colour (present/ absent; CmC-dimorphism), diet (browsers/ grazers/ generalist; CmC-feeding)or no partition (null model M2a-rel; Weadick and Chang 2010). The significance (*) of each model is tested against the null model by LRT after correcting for multiple testing (FDR=0.05) and the relative fit of different models compared by AIC. Values of the coefficient of selection are shown for the two assumed taxa partitions (background/foreground) indicating their phenotype. Analyses performed with PAML clade C models, which assume three site classes: class 0 (negatively selected), class 1 (neutral sites; not shown) and class 2 (variable).

|  |  |  |  | LRT |  | Site class 0 | |  | Site class 2 ^a^ | | | | | | | |  |
| --- | --- | --- | --- | --- | --- | --- | --- | --- | --- | --- | --- | --- | --- | --- | --- | --- | --- |
| Opsin gene | Model tested | *ln*L | ∂AIC | 2∆*ln*L |  | ω | *p* |  | Background ω b | | Foreground ω | | | *p* | | | |
| SWS1 | CmC-Tropheini | -2142.033 | 0 | 14.163* |  | 0 | (0.615) |  | basal.: | 17.301 | Tropheini: | 0 | | (0.016) | | |  |
|  | CmC-feeding | -2142.033 | 2.000 | 14.163* |  | 0 | (0.615) |  | omnivorous | 3.970 | browsers: | 0 | | (0.016) | | |  |
|  |  |  |  |  |  |  |  |  |  |  | grazers: | | 0 | |  |  |  |
|  | CmC-dimorphism | -2147.064 | 10.062 | 4.102^NS^ |  | 0 | (0.622) |  | m.c.: | 0 | d.c.: | 8.305 | | (0.049) | | |  |
|  | M2a-rel | -2149.115 | 12.163 |  |  | 0 | (0.620) |  |  | 8.251 |  |  | | (0.014) | | |  |
| SWS2B | M2a-rel | -2095.170 | 0 |  |  | 0 | (0.658) |  |  | 11.241 |  |  | | (0.035) | | |  |
|  | CmC-dimorphism | -2094.983 | 1.625 | 0.375^NS^ |  | 0 | (0.802) |  | m.c.: | 16.758 | d.c.: | 9.549 | | (0.049) | | |  |
|  | CmC-Tropheini | -2095.170 | 1.999 | 0.001^NS^ |  | 0 | (0.657) |  | basal.: | 11.164 | Tropheini: | 11.341 | | (0.035) | | |  |
|  | CmC-feeding | -2094.477 | 2.613 | 1.387^NS^ |  | 0 | (0.652) |  | omnivorous | 11.984 | browsers: | 8.340 | | (0.032) | | |  |
|  |  |  |  |  |  |  |  |  |  |  | grazers: | 20.006 | |  | | |  |
| SWS2A | CmC-Tropheini | -1836.923 | 0 | 28.074* |  | 0 | (0.744) |  | basal.: | 0 | Tropheini: | 133.041 | | (0.008) | | |  |
|  | CmC-feeding | -1836.077 | 0.308 | 29.766* |  | 0 | (0.744) |  | omnivorous | 0 | browsers: | 179.864 | | (0.008) | | |  |
|  |  |  |  |  |  |  |  |  |  |  | grazers: | 31.430 | |  | | |  |
|  | CmC-dimorphism | -1843.327 | 12.809 | 15.265* |  | 0 | (0.746) |  | m.c.: | 999 | d.c.: | 2.820 | | (0.006) | | |  |
|  | M2a-rel | -1850.960 | 26.074 |  |  | 0 | (0.766) |  |  | 14.154 |  |  | | (0.018) | | |  |
| RH2B | CmC-Tropheini | -1290.946 | 0 | 7.935* |  | 0 | (0.677) |  | basal.: | 10.162 | Tropheini: | 0 | | (0.120) | | |  |
|  | CmC-feeding | -1290.946 | 2.000 | 7.935^NS^ |  | 0 | (0.677) |  | omnivorous | 10.166 | browsers: | 0 | | (0.120) | | |  |
|  |  |  |  |  |  |  |  |  |  |  | grazers: | 0 | |  | | |  |
|  | M2a-rel | -1294.913 | 5.935 |  |  | 0 | (0.745) |  |  | 1.993 |  |  | | (0.254) | | |  |
|  | CmC-dimorphism | -1293.981 | 6.069 | 1.865^NS^ |  | 0 | (0.672) |  | m.c.: | 0 | d.c.: | 2.613 | | (0.177) | | |  |
| LWS | CmC-Tropheini | -2086.000 | 0 | 26.900* |  | 0.038 | (0.911) |  | basal.: | 14.551 | Tropheini: | 0.715 | | (0.089) | | |  |
|  | CmC-feeding | -2086.092 | 2.184 | 26.716* |  | 0.040 | (0.912) |  | omnivorous | 14.678 | browsers: | 1.083 | | (0.088) | | |  |
|  |  |  |  |  |  |  |  |  |  |  | grazers: | 0 | |  | | |  |
|  | M2a-rel | -2099.450 | 24.900 |  |  | 0 | (0.826) |  |  | 8.048 |  |  | | (0.066) | | |  |
|  | CmC-dimorphism | -2099.086 | 26.172 | 0.728^NS^ |  | 0 | (0.835) |  | m.c.: | 0 | d.c.: | 7.986 | | (0.071) | | |  |

^a^ Abbreviations refer to sexually monomorphic in coloration (m.c.), sexually dimorphic in coloration (d.c.).
