# Supplementary material for: Phylogenomics of trophically diverse cichlids disentangles processes driving adaptive radiation and repeated trophic transitions
Source: Ecol Evol. 2022 Jul 17;12(7):e9077. doi: 10.1002/ece3.9077 (PMC9288888; doi:10.1002/ece3.9077)
Supplement: Supplementary file 6 — File S4 [file ECE3-12-e9077-s007.zip › ece39077-sup-0004-FileS4A.docx]

Singh et al

**Set 1: Opsins** (Carleton 2009)

LWS: Long wave sensitive opsin. Red sensitivity (6 exons)

SWS1: Short wave sensitive opsin 1- UltraViolet sensitivity (5 eons)

SWS2a: Short wave sensitive opsin 2a - Blue sensitivity (5 exons)

SWS2b: Short wave sensitive opsin 2b - Violet sensitivity (5 exons)

RH2B: Mid wave sensitive opsin. Rhodopsin like 2b - Green-blue sensitivity (5 exons)

RH2A-alpha: Mid wave sensitive opsin. Rhodopsin like 2A-alpha - Green sensitivity (5 exons)

RH2A-beta: Mid wave sensitive opsin. Rhodopsin like 2A-beta - Green sensitivity (5 exons)

RH1: Mid wave sensitive opsin. Rhodopsin - Twilight sensitivity (5 exons)

**Set 2: color genes**

CSF1ra: under positive selection in Haplochromines, xantophores, eggspots (Salzburger *et al.* 2007)

DLC: Stripe patterning in Zebrafish, melanophore-xantophore interaction (Hamada *et al.* 2014)

FBXO36b: Positional candidate for stripe patterning in *Haplochromis sauvagei* (Henning *et al.* in press)

HAGOROMO: Stripe patterning in Zebrafish (Kawakami *et al.* 2000). Under positive selection in Haplochromines (Terai *et al.* 2002), increased splicing diversity in haplochromines (Terai *et al.* 2003),

KIR7.1: Stripe patterning in Zebrafish (Inaba *et al.* 2012; Singh *et al.* 2014)

KIR7.2: Cichlid specific duplicate, evidence for neofunctialization (Watanabe *et al.* 2007)

KITa: Melanophore developlment and proliferation in zebrafish (Parichy *et al.* 1999)

KITLa: Melanophore developlment and proliferation in stickleback and humans (Miller *et al.* 2007)

MITFa: Melanophore survival and viability in mice (Tachibana *et al.* 1994) and human disease models (Tassabehji *et al.* 1994; McGill *et al.* 2002)

SMTLb: Proliferation and morphogenesis of pigment cells and mating preference in Medaka (Fukamachi *et al.* 2004; Fukamachi *et al.* 2009a; Fukamachi *et al.* 2009b)

SOX10: Pigment cell proliferation and viability in zebrafish (Dutton *et al.* 2001)

**Set 3: pharyngeal jaw apparatus**

BARX1. Involved in dental regulatory circuit; expressed in cichlid LPJ (Fraser *et al.* 2009).

BMP2. Involved in odontegenesis in mice (Fraser *et al.* 2009); Upregulated in hard diet (Gunter *et al.* 2013), gene from osteoblast proliferation/differentiation pathway. Early neurocrest inducer (Betancur *et al.* 2010).

BMP4. Involved in odontegenesis in mice (Fraser *et al.* 2009); craniofacial development (Helms *et al.* 2005); Early neurocrest inducer (Betancur et al. 2010 Ann Rev Cell Dev Biol).

COL6A1. Collagen gene, important in development of bone; Expressed in cichlid lower pharyngeal jaw (Schneider et al. *in press*).

CREB1. Transcription factor with binding sites in gene network from Schneider et al. 2014 Mol Ecol.

DLX2. Involved in dental regulatory circuit; expressed in cichlid LPJ (Fraser *et al.* 2009).

C-FOS. Transcription factor with binding sites in gene network from Schneider et al. (*in press*).

PITX2. Involved in dental regulatory circuit; expressed in cichlid LPJ (Fraser *et al.* 2009).

RUNX2B. Involved in odontegenesis in mice (Fraser *et al.* 2009); Upregulated in hard diet (Gunter *et al.* 2013), gene from osteoblast proliferation/differentiation pathway.

RX1. Only homolog of human prrx2 in tilapia. Craniofacial bone development in mice (Lu et al. 1999); TF with many biding sites in genes from Schneider et al. (*in press)*

SHH. Involved in dental regulatory circuit; expressed in cichlid LPJ (Fraser *et al.* 2009).

SP7. (=osx) Gene of extracellular matrix. Upregulated in hard diet (Gunter *et al.* 2013), gene from osteoblast proliferation/differentiation pathway.

**References**

Betancur P, Bronner-Fraser M, Sauka-Spengler T (2010) Assembling Neural Crest Regulatory Circuits into a Gene Regulatory Network. *Annual Review of Cell and Developmental Biology, Vol 26* **26**, 581-603.

Carleton K (2009) Cichlid fish visual systems: mechanisms of spectral tuning. *Integrative Zoology* **4**, 75-86.

Dutton KA, Pauliny A, Lopes SS*, et al.* (2001) Zebrafish colourless encodes sox10 and specifies non-ectomesenchymal neural crest fates. *Development* **128**, 4113-4125.

Fraser GJ, Hulsey CD, Bloomquist RF*, et al.* (2009) An Ancient Gene Network Is Co-opted for Teeth on Old and New Jaws. *PLoS Biology* **7**, 233-247.

Fukamachi S, Kinoshita M, Aizawa K*, et al.* (2009a) Dual control by a single gene of secondary sexual characters and mating preferences in medaka. *BMC Biology* **7**, 64.

Fukamachi S, Sugimoto M, Mitani H, Shima A (2004) Somatolactin selectively regulates proliferation and morphogenesis of neural-crest derived pigment cells in medaka. *Proceedings of the National Academy of Sciences of the United States of America* **101**, 10661-10666.

Fukamachi S, Yada T, Meyer A, Kinoshita M (2009b) Effects of constitutive expression of somatolactin alpha on skin pigmentation in medaka. *Gene* **442**, 81-87.

Gunter HM, Fan SH, Xiong F*, et al.* (2013) Shaping development through mechanical strain: the transcriptional basis of diet-induced phenotypic plasticity in a cichlid fish. *Molecular Ecology* **22**, 4516-4531.

Hamada H, Watanabe M, Lau HE*, et al.* (2014) Involvement of Delta/Notch signaling in zebrafish adult pigment stripe patterning. *Development* **141**, 318-324.

Helms JA, Cordero D, Tapadia MD (2005) New insights into craniofacial morphogenesis. *Development* **132**, 851-861.

Henning F, Lee H, Franchini P, Meyer A (in press) Genetic mapping of horizontal stripes in Lake Victoria cichlid fishes: benefits and pitfalls of using of dense linkage mapping in non-model organisms. *Molecular Ecology*.

Inaba M, Yamanaka H, Kondo S (2012) Pigment pattern formation by contact-dependent depolarization. *Science* **335**, 677-677.

Kawakami K, Amsterdam A, Shimoda N*, et al.* (2000) Proviral insertions in the zebrafish hagoromo gene, encoding an F-box/WD40-repeat protein, cause stripe pattern anomalies. *Current Biology* **10**, 463-466.

McGill GG, Horstmann M, Widlund HR*, et al.* (2002) Bcl2 regulation by the melanocyte master regulator Mitf modulates lineage survival and melanoma cell viability. *Cell* **109**, 707-718.

Miller CT, Beleza S, Pollen AA*, et al.* (2007) cis-regulatory changes in *kit ligand* expression and parallel evolution of pigmentation in sticklebacks and humans. *Cell* **131**, 1179-1189.

Parichy DM, Rawls JF, Pratt SH, Whitfield TT, Johnson SL (1999) Zebrafish sparse corresponds to an orthologue of c-kit and is required for the morphogenesis of a subpopulation of melonocytes but is not essential for hematopoises or primordial germ cell development. *Development* **126**, 3425-3436.

Salzburger W, Braasch I, Meyer A (2007) Adaptive sequence evolution in a color gene involved in the formation of the characteristic egg-dummies of male haplochromine cichlid fishes. *BMC Biology* **5**, 1.

Singh AP, Schach U, Nusslein-Volhard C (2014) Proliferation, dispersal and patterned aggregation of iridophores in the skin prefigure striped colouration of zebrafish. *Nature Cell Biology* **16**, 604-+.

Tachibana M, Perezjurado LA, Nakayama A*, et al.* (1994) Cloning of Mitf, the Human Homolog of the Mouse Microphthalmia Gene and Assignment to Chromosome 3p14.1 - P12.3. *Human Molecular Genetics* **3**, 553-557.

Tassabehji M, Newton VE, Read AP (1994) Waardenburg Syndrome Type-2 Caused by Mutations in the Human Microphthalmia (Mitf) Gene. *Nature Genetics* **8**, 251-255.

Terai Y, Morikawa N, Kawakami K, Okada N (2002) Accelerated evolution of the surface amino acids in the WD-repeat domain encoded by the hagoromo gene in an explosively speciated lineage of east African cichlid fishes. *Molecular Biology and Evolution* **19**, 574-578.

Terai Y, Morikawa N, Kawakami K, Okada N (2003) The complexity of alternative splicing of hagoromo mRNAs is increased in an explosively speciated lineage in East African cichlids. *Proceedings of the National Academy of Sciences of the United States of America* **100**, 12798-12803.

Watanabe M, Hiraide K, Okada N (2007) Functional diversification of kir7.1 in cichlids accelerated by gene duplication. *Gene* **399**, 46-52.
